# Carbon Nanofiber/Polyaniline Composite Aerogel with Excellent Electromagnetic Interference Shielding, Low Thermal Conductivity, and Extremely Low Heat Release

**DOI:** 10.1007/s40820-024-01583-2

**Published:** 2024-12-02

**Authors:** Mingyi Chen, Jian Zhu, Kai Zhang, Hongkang Zhou, Yufei Gao, Jie Fan, Rouxi Chen, Hsing-Lin Wang

**Affiliations:** 1 School of Textile Science and Engineering, Tiangong University, Tianjin, 300387 People’s Republic of China; 2 Department of Materials Science and Engineering, Southern University of Science and Technology, Shenzhen, 518055 People’s Republic of China; 3 School of Innovation and Entrepreneurship, Southern University of Science and Technology, Shenzhen, 518055 People’s Republic of China; 4 Guangdong Provincial Key Laboratory of Energy Materials for Electric Power, Southern University of Science and Technology, Shenzhen, 518055 People’s Republic of China; 5 Ministry of Education Key Laboratory of Advanced Textile Composite Materials, Tiangong University, Tianjin, 300387 People’s Republic of China

**Keywords:** CNFs/PANI hybrid aerogels, EMI shielding, Flame retardancy, Thermal insulation

## Abstract

The rapid development of communication technology and high-frequency electronic devices has created a need for more advanced electromagnetic interference (EMI) shielding materials. In response to this demand, a study has been conducted to develop multifunctional carbon nanofibers (CNFs)/polyaniline (PANI) aerogels with excellent electromagnetic interference shielding, flame retardancy, and thermal insulation performance. The process involved freeze-drying of electrospun CNFs and PANI nanoparticles followed by in situ growth PANI to coat the CNFs, creating the core–shell structured CNFs/PANI composite fiber and its hybrid aerogels (CP-3@PANI). The interaction between PANI and aniline (ANI) provides attachment sites, allowing additional ANI adsorption into the aerogel for in situ polymerization. This results in PANI uniformly covering the surface of the CNFs, creating a core–shell composite fiber with a flexible CNF core and PANI shell. This process enhances the utilization rate of the ANI monomer and increases the PANI content loaded onto the aerogel. Additionally, effective connections are established between the CNFs, forming a stable, conductive three-dimensional network structure. The prepared CP-3@PANI aerogels exhibit excellent EMI shielding efficiency (SE) of 85.4 dB and specific EMI SE (SE d^−1^) of 791.2 dB cm^3^ g⁻^1^ in the X-band. Due to the synergistic flame-retardant effect of CNFs, PANI, and the dopant (phytic acid), the CP-3@PANI aerogels demonstrate outstanding flame-retardant and thermal insulation properties, with a peak heat release rate (PHRR) as low as 7.8 W g⁻^1^ and a total heat release of only 0.58 kJ g⁻^1^. This study provides an effective strategy for preparing multifunctional integrated EMI shielding materials.

**Supplementary Information:**

The online version contains supplementary material available at 10.1007/s40820-024-01583-2.

## Highlights

- Using electrospun high-strength carbon nanofibers as the aerogel scaffold, high loading of polyaniline in the aerogel was achieved through seed polymerization, forming the core–shell structure fibers and simultaneously connecting the fibers as a stable conductive network.
- Combining carbon-based materials with conductive polymers like polyaniline significantly improves shielding performance, achieving electromagnetic interference shielding efficiency of up to 84.5 dB and SE d^−1^ values greater than 791.2 dB cm^3^ g⁻^1^. Flame retardancy reduces PHRR by 65.8%, and thermal insulation with low thermal conductivity of 0.104 W m⁻^1^ K⁻^1^.

**Supplementary Information:**

The online version contains supplementary material available at 10.1007/s40820-024-01583-2.

## Graphical abstract

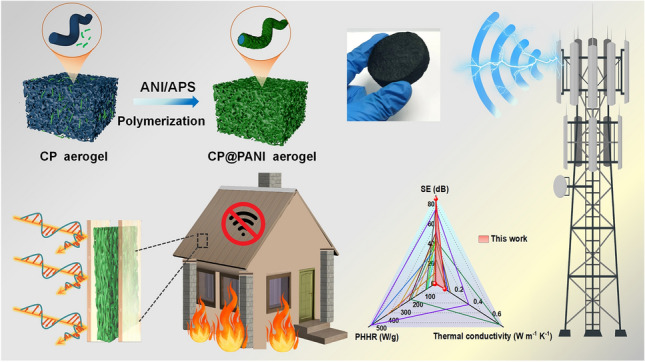

## Introduction

The rapid development and widespread adoption of 5G technology and millimeter wave technology have significantly improved communication speed and efficiency. However, these advancements also come with challenges, particularly regarding electromagnetic interference (EMI) and electromagnetic radiation pollution. This affects the operation of precision devices and poses significant health risks. Consequently, EMI shielding materials have garnered considerable attention to address this critical issue effectively. Researchers have primarily concentrated on improving the efficiency of shielding and reducing the weight and thickness of EMI shielding materials for potential use in civilian and military equipment. Various nanostructured materials, such as carbon nanotubes (CNTs) [1], graphene [2, 3], transition metal carbides [4], metal nanowires [5], and conductive polymers [6], have been widely used in the field of EMI shielding. Despite these advancements, most previous research has mainly focused on enhancing EMI shielding performance. However, future applications of EMI shielding materials may extend to more complex real-world scenarios, necessitating multifunctional materials to meet application requirements. Absorbing electromagnetic waves (EMW) often generates heat, which can cause structural degradation and pose safety hazards. Consequently, EMI shielding materials that are flame-retardant and thermally insulating are essential, as they can effectively mitigate indoor EMI and pollution while preventing hazards to buildings from the heat generated by EMW absorption [7, 8]. Moreover, flexible and flame-retardant EMI shielding materials are highly sought after for wearable electronic devices, where high flame resistance can prevent thermal runaway and fire risks from prolonged operation [9]. Thermal insulation is also an essential feature for EMI shielding materials, as it can protect targets from heat/cold damage and provide infrared stealth capabilities by reducing thermal conductivity, thereby protecting devices from infrared detection [10].

Lin et al. [8] developed a multifunctional nano-/micro-coating on commercial polyurethane (PU) foam by in situ growth of PANI and dip coating of silver nanowires, successfully constructing a unique continuous micro-/nanostructure on the PU foam surface, resulting in excellent flame-retardant and EMI shielding properties. In Gong et al. [11] a multifunctional flexible PCC/MXene/PVA phase-change composite film was prepared via a one-step vacuum-assisted filtration method, which exhibited high photothermal conversion efficiency, Joule heating, fire safety, and EMI shielding effects. Shi et al. [12] synthesized a novel multifunctional melamine-based hybrid foam through co-precipitation and dip coating processes, retaining high thermal insulation in addition to EMI shielding performance due to its multilayered porous structure. Therefore, combining flame retardancy, thermal insulation, and EMI shielding offers broader prospects for civilian and military applications compared to single-function EMI shielding materials [13]. Achieving multifunctionality in EMI shielding materials is a future development direction. However, the complexity of integrating multifunctional devices and the scarcity of multifunctional nanomaterials make such materials rare, presenting a significant challenge in preparing multifunctional integrated EMI shielding materials.

Carbon nanofibers (CNFs), due to their lightweight nature, high specific surface area, corrosion resistance, and excellent thermal stability, have found widespread use in EMI shielding applications [14, 15]. The material's development aligns with sustainability goals by incorporating a low-carbon manufacturing process and utilizing sustainable materials with a negative carbon footprint, significantly reducing its environmental impact in the context of 5G technology and its associated infrastructure [16]. To render CNFs suitable for advanced EMI shielding systems, researchers have introduced conductive and magnetic fillers to enhance the EMI shielding performance of the materials [17]. Recently, combining carbon-based materials with intrinsically conductive polymers (ICP) to form synergistic carbon nanocomposites has gained research, particularly with polyaniline (PANI) due to its superior electrochemical properties, facile synthesis, strong conductivity, and environmental stability. For example, Kumar et al. [18] supplemented high apparent density vapor-grown carbon fibers (VGCNFs-H) as conductive fillers to prepare PANI-based hybrid nanocomposites, exhibiting 51 dB EMI shielding efficiency (SE) in the X-band. Javaria et al. [19] achieved a total EMI SE of 16.80 dB by decorating carbon nanotubes with cobalt ferrite nanoparticles and coating them with PANI. Das et al. [20] increased the EMI SE of a paraffin-based composite to 22.5 dB by chemically oxidizing aniline (ANI) on sulfur-doped reduced graphene oxide (RGO) surfaces. Xing et al. [21] created a hierarchical carbon nanotube-coated cotton fabric/polyaniline (CNT-CC/PANI) nanocomposite by growing carbon nanotubes (CNT) on cotton fabric through a single-step carbonization and in situ polymerizing PANI nanowires, achieving over 40 dB EMI SE. Xie et al. [22] synthesized a ternary film with an EMI SE of 78 dB at 10 GHz by combining suction filtration of multiwalled carbon nanotube/graphene (MWCNT/Gr) films and electrochemical deposition of PANI on MWCNT/Gr films.

Recent studies have shown that the structure of shielding materials (e.g., sandwich, multilayer, and foam structures) significantly impacts their EMI shielding performance [23–25]. Researchers are exploring structural engineering strategies to design complex microstructures to address issues such as material thickness, weight, and material loss. Aerogels are gaining attention due to their large surface area and rich porous structure, which effectively enhances internal multiple reflections and scattering of EMW to attenuate energy [26–28]. Additionally, aerogels typically offer excellent thermal insulation due to their high porosity, three-dimensional network skeleton, and ultra-low thermal conductivity from high gas content, achieving multifunctional integrated performance [29].

In this work, we have synthesized carbon nanofiber-based aerogels with outstanding flame retardancy, thermal insulation, and high electromagnetic shielding performance. The high-strength electrospun polybenzimidazole-dihydroxy-diketone (BBB)-derived CNFs, carboxymethyl cellulose, and PANI were used as the framework, binder, and the conductive bridge, respectively, to prepare the hybrid aerogels through freeze-drying. We prepared hybrid aerogels through freeze-drying. The aerogels were soaked in an ANI solution, utilizing hydrogen bonding between PANI and ANI to adsorb more ANI into the aerogel for in situ polymerization. The in situ polymerized PANI uniformly covered the surface of the CNFs. Additionally, the PANI coating bridges any disruptions caused by nanocellulose, enhancing overall conductivity and forming a stable, conductive three-dimensional network structure. This core–shell structure design enhances the dissipation capability of the composite aerogel for EMW through interface polarization, multiple reflections, and scattering. We investigated the effects of PANI content on the core–shell structure, electromagnetic shielding performance, flame retardancy, and aerogel thermal insulation. The prepared CP-3@PANI aerogel exhibited optimal EMI SE of up to 84.5 dB, with SE d^−1^ greater than 791.2 dB cm^3^ g⁻^1^, surpassing most PANI-based and carbon fiber-based aerogels. Under the ternary synergistic effect of CNFs, PANI, and PA, CP-3@PANI aerogel demonstrated excellent flame retardancy and thermal insulation, with PHRR and THR reduced by 65.8% and 64.8%, respectively, compared to pure CNFs aerogel. Therefore, the CP-3@PANI aerogel provides a new strategy for developing and utilizing materials for building protective layers and civilian or military applications.

## Experimental and Calculation

### Materials

Polyvinylpyrrolidone (PVP) with a molecular weight of 1,300,000, 1,4,5,8-naphthalene tetracarboxylic acid (NTCA), 3,3'-diaminobenzidine (DAB), aniline (ANI), N, N-dimethylformamide (DMF), and dodecylbenzene sulfonic acid (DBSA) were purchased from Macklin Biochemical Co., Ltd., Shanghai, China. Ammonium persulfate (APS), concentrated hydrochloric acid (HCl), d-camphorsulfonic acid (CSA), 50% phytic acid (PA), and ammonia solution (NH_3_·H_2_O) were obtained from Aladdin Reagent Co., Ltd., Shanghai, China. The nanocellulose (a diameter of 10–20 nm and a length of 5–10 μm) suspension (containing 1.5 wt% nanocellulose) was purchased from Tianjin Woodelf Biotechnology Co., Ltd., Tianjin, China. All reagents were of analytical grade and were used without further purification.

### Preparation of BBB-Based CNFs

First, 0.36 g of PVP was dissolved in 1.44 g of DMF, stirred on a heating platform at 60 °C for 8 h until fully dissolved, and then allowed to cool. This PVP solution was used as the co-spinning agent for electrospinning. Next, 0.49 g of NTCA and 0.35 g of DAB were added to 3.36 g of DMF and stirred at 20 °C for 24 h to create the NTCA-DAB solution. The PVP and NTCA-DAB solutions were mixed and stirred for 4 h before being used for electrospinning to obtain NTCA-DAB-PVP nanofiber membranes. The NTCA-DAB-PVP nanofibers were heat-treated at 450 °C for 1 h and then carbonized in an argon atmosphere at 1200 °C. The heating rate during carbonization was set to 8 °C per minute. Finally, the material was annealed at 1200 °C for 1 h to complete the carbonization process, forming BBB-derived CNF membranes.

### Preparation of CNFs Aerogel and CNFs/PANI Hybrid Aerogel

0.32 g of CNF membranes and 2 g of deionized water were added to a grinder (equipped with four blades and operating at a speed of 22,000 RPM) and ground for 5 min. After thorough grinding, the CNFs exhibited a length distribution of 50–1000 μm (Fig. S1). Subsequently, 4 g of nanocellulose was added and thoroughly mixed, acting as a binder to facilitate the formation of the aerogel’s network structure. The mixture was then poured into a mold and frozen for 24 h, followed by freeze-drying for 72 h to obtain the CNFs aerogel, designated as CFA. PANI synthesis is carried out following a recently reported method [6]. 4.0 g of aniline was dissolved in 50 mL of 1 M hydrochloric acid solution, and 12.3 g of ammonium persulfate was also dissolved in 50 mL of 1 M hydrochloric acid solution. The two solutions were mixed at room temperature for 6 h. The concentration of the reactants was 0.2 M aniline and 0.25 M ammonium persulfate. CNFs and PANI were mixed in mass ratios of 1:1, 1:2, and 1:3, respectively, while maintaining the same conditions as the CNFs aerogel. The resulting hybrid aerogels were designated as CP-1, CP-2, and CP-3. Detailed mass ratios are provided in Table S1.

### Preparation of CF@PANI, CP-1@PANI, CP-2@PANI, and CP-3@PANI Aerogels

The following process was utilized to fabricate core–shell structured aerogels using CNFs and CNFs/PANI aerogels as substrates. A mixture of 450 mL deionized water, 450 mL anhydrous ethanol, and 50 mL phytic acid aqueous solution was prepared and divided equally into Solution A and Solution B. 1.0 g of ANI was added dropwise into Solution A and mixed thoroughly. The aerogel was then immersed in Solution A and allowed to stand for 2 h. Subsequently, 2.45 g of APS was added to Solution B and sonicated for 2 min to ensure complete dissolution and then mixed with Solution A. The reactor was placed in a 0 °C ice water bath for in situ polymerization for 12 h. After polymerization, the aerogels were removed, washed with ethanol and deionized water, and freeze-dried for 72 h to obtain core–shell structured aerogels. Using this method, CNFs@PANI (named as CF@PANI), CP-1@PANI, CP-2@PANI, and CP-3@PANI aerogels were prepared.

### Acid Treatment of CP-3@PANI Aerogels

After being soaked and rinsed in a basic solution, PANI can be developed to its intrinsic state, which can then be doped with proton acids. The procedure is as follows: CP-3@PANI aerogels were added to 100 mL of 0.1 M ammonia solution and sonicated for 3 min to ensure complete dedoping of PANI. The aerogels were then rinsed with deionized water and vacuum-dried at 60 °C for 24 h. Next, the CP-3@PANI aerogels were immersed in 100 mL of 0.1 M HCl, CSA, and DBSA solutions for acid doping by soaking for 12 h. Finally, the aerogels were freeze-dried for 72 h to obtain acid-doped CP-3@PANI aerogels. These samples were used to compare the EMI shielding performance of CP-3@PANI aerogels doped with different acids.

### Comprehensive Characterization and Performance Evaluation

The samples were examined using a scanning electron microscope (SEM, ESCAN VEGA3 LMH) and transmission electron microscopy (TEM) (HT-7700, Holland, power at 5 kV) with energy-dispersive X-ray spectroscopy (EDS) to analyze their surface morphology and elemental composition. The crystallinity of all samples was examined by X-ray diffraction (XRD, D8-Discover, Bruker, Karlsruhe, Germany). The samples were positioned on a test slide groove and scanned from 5° to 45° at a rate of 0.1° per second to measure the diffraction as a function of the angle. The thermal stability of the composite aerogels was determined using a thermogravimetric analyzer (TG 209F3, NETZSCH, Bavaria, Germany) with a gas flow rate set to 50 mL min⁻^1^. The temperature range was from 25 to 800 °C with a heating rate of 10 °C min⁻^1^. Samples were held at 105 °C for 2 min to remove moisture, then cooled to 25 °C at 10 °C min⁻^1^, and subsequently heated to 800 °C at 10 °C min⁻^1^ to observe mass loss as a function of temperature. X-ray photoelectron spectroscopy (XPS) measurements were conducted using an XPS (XSAM80 Kratos Co, UK) with Al Kα excitation radiation (1486.6 eV). Raman spectra were measured using a confocal Raman microscope (CRM) (Alpha300R, WITec GmbH, Germany) equipped with a TEM single-frequency laser (λ = 532 nm, laser power = 40 mW). The specific surface area was measured using the Brunauer–Emmett–Teller (BET) method with a Micromeritics ASAP 2020 (USA). The pore diameter and pore volume were determined based on the Barrett–Joyner–Halenda (BJH) theory, utilizing the adsorption branch of the isotherm data. The contact angle (CA) of the sample was tested using an optical CA measuring device (VCA optima, AST Products, INC). The combustion behavior of the aerogels was studied using a microscale combustion calorimeter (MCC) (Fire Testing Technology Ltd., East Grinstead, UK) according to ASTM D7309-2007 standard. The samples (~ 5.0 mg) were heated from 75 to 750 °C at a heating rate of 1 °C s⁻^1^ under a mixed flow of N_2_ (80%) and O_2_ (20%). The limited oxygen index (LOI) values were measured with an oxygen index meter (ATSFAAR 2008600, Italy) based on ASTM D2863‐2009. The dimensions of tested samples were 120 × 10 × 10 mm^3^. Thermal conductivity was measured using a DRE-III-X thermal conductivity tester (Xiangtan Xiangyi Instrument Co., Ltd., China). Each sample was tested three times, and the recorded value was the average of the three tests. The aerogels' apparent density (ρ, mg cm⁻^3^) was determined by dividing the mass by the volume of the aerogel. The conductivity of the sample was measured at room temperature using a four-probe tester (RTS-4, Beijing Shichuang Technology, China). The porosity ($\uprho$) of CP-3@PANI was calculated using the formula:

$$ P\left( \% \right) = \left( {1 - \rho /\rho_{solid} } \right) \times 100\% , $$

where $\rho$ is the density of CP-3@PANI, and ${\rho }_{solid}$ is the density of the CNFs/PANI aerogel.

EMI SE and EM parameters were measured using an Agilent E5071C vector network analyzer by the wave guide method; samples have a thickness of 4.5 mm. The measured scattering parameters (Figs. S11 and S21) were used to calculate the absorption, reflection, and total shielding values. EMI SE (SE_T_) is divided into three components: reflection loss (SE_R_), absorption loss (SE_A_), and multiple reflection loss (SE_M_), and the formula for calculating each element can be seen in the first part of the supporting information.

## Results and Discussion

### Chemical Structure and Nanofiber Morphology

**Fig. 1:**
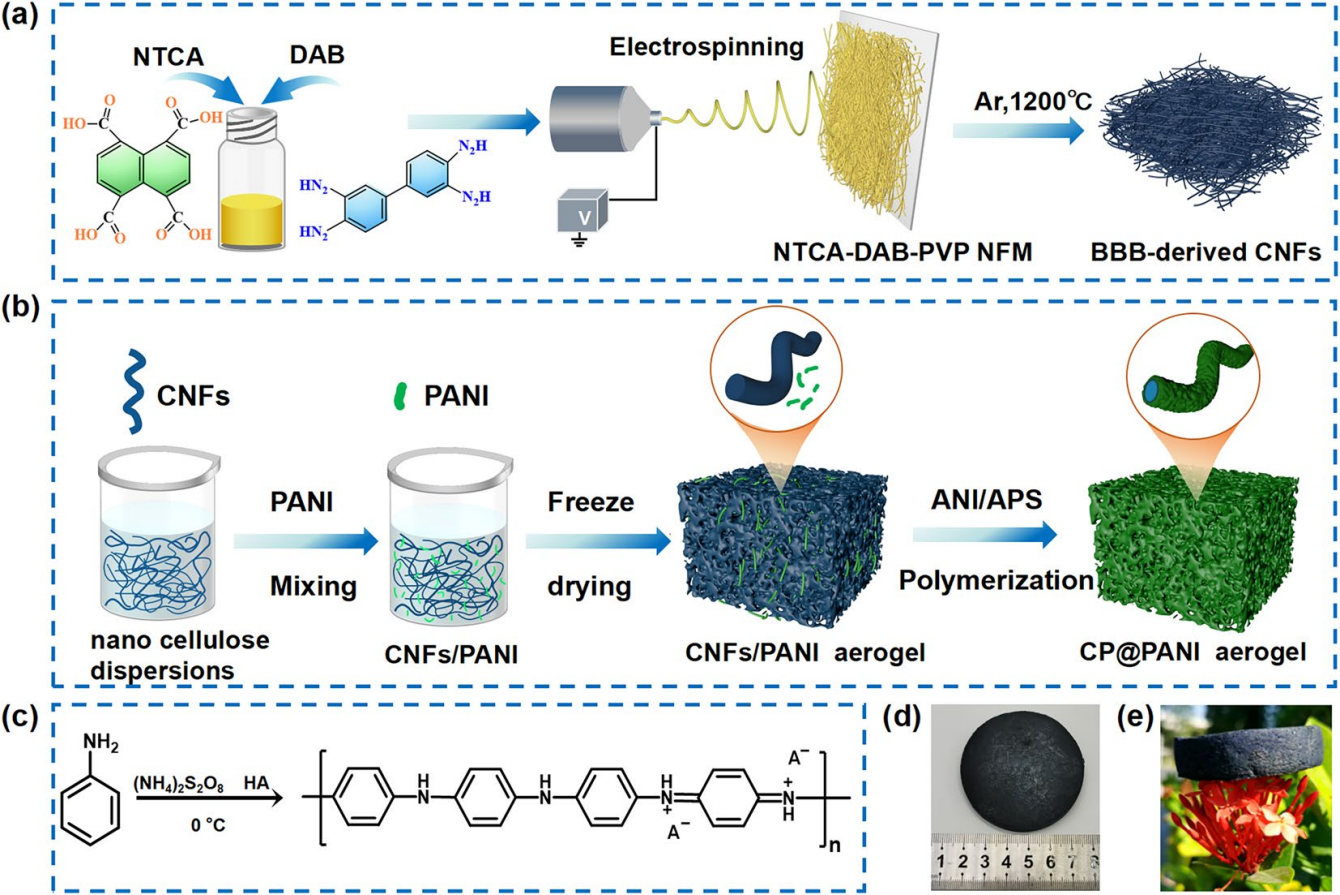
**a** Schematic illustration of the preparation of BBB nanofibers and their derived carbon fiber membranes. **b** Schematic illustration of the preparation method of CP-3@PANI aerogels. **c** Chemical structure of the product after oxidative polymerization. **d** Photograph of the CP-3@PANI aerogel. **e** Photograph demonstrating the lightweight nature of the CP@PANI aerogel

Figure 1a illustrates the synthetic scheme of BBB nanofibers and their derived CNF membranes. Specifically, NTCA and DAB were stirred in DMF at low temperatures with the addition of a sacrificial additive polymer, PVP, to obtain a homogeneous spinning solution [30]. Continuous, smooth, and uniform precursor nanofibers with an average diameter of 900 nm (Fig. S2a) were obtained by electrospinning. The precursor nanofiber membranes were heat-treated at 450 °C for 1 h to obtain BBB nanofibers, followed by annealing at 1200 °C for 1 h to complete the carbonization process, resulting in BBB-derived CNFs membranes (Figs. S3 and S4). The as-prepared CNFs maintain this smooth cylindrical shape, with their diameter reduced to 580 nm (Fig. S2b), exhibiting both high strength and exceptional mechanical flexibility (Fig. S5). Figure 1b shows the process of preparing CNFs/PANI@PANI composite aerogels, denoted as CP@PANI. The CNFs were ground into short fibers, dispersed in deionized water, and mixed with synthesized PANI nanoparticles (Fig. S6), and a suitable amount of nanocellulose was added as a binder. The mixture was then freeze-dried to achieve CNFs and CNFs/PANI hybrid aerogels. As shown in Fig. S7, nanocellulose entangles with the CNFs and serves as junctions to help form a stable structure. The prepared aerogels were soaked in an aniline (ANI) solution containing phytic acid and APS. The hydrogen bonding between PANI and ANI facilitated the adsorption of more ANI onto the aerogel surface for in situ polymerization (reaction equation shown in Fig. 1c). The CFA@PANI exhibited an uneven PANI coating on the CNF surface, increasing the fiber diameter to 940 nm (Fig. S2c). When mixed with PANI in a 1:1 weight ratio (CP-1@PANI), the PANI coating becomes uniform, and the fiber diameter reaches 950 nm (Fig. S2d). For the 1:2 (CP-2@PANI) and 1:3 (CP-3@PANI) ratios, the fiber diameters increase to 1.1 and 1.19 mm, respectively, with consistent uniform coatings (Fig. S2e, f). The surface of the carbon nanofibers is evenly covered with in situ polymerized PANI, which connects the CNFs to form a stable, conductive three-dimensional network structure. The resulting CP-3@PANI aerogels are lightweight and structurally stable, as depicted in Fig. 1d, e. During immersion in the solution, PANI undergoes doping with PA, resulting in a deep green coloration of the aerogels. This same deep green hue is also evident in the precipitated PANI particles formed after polymerization in the solution (Fig. S8).

**Fig. 2:**
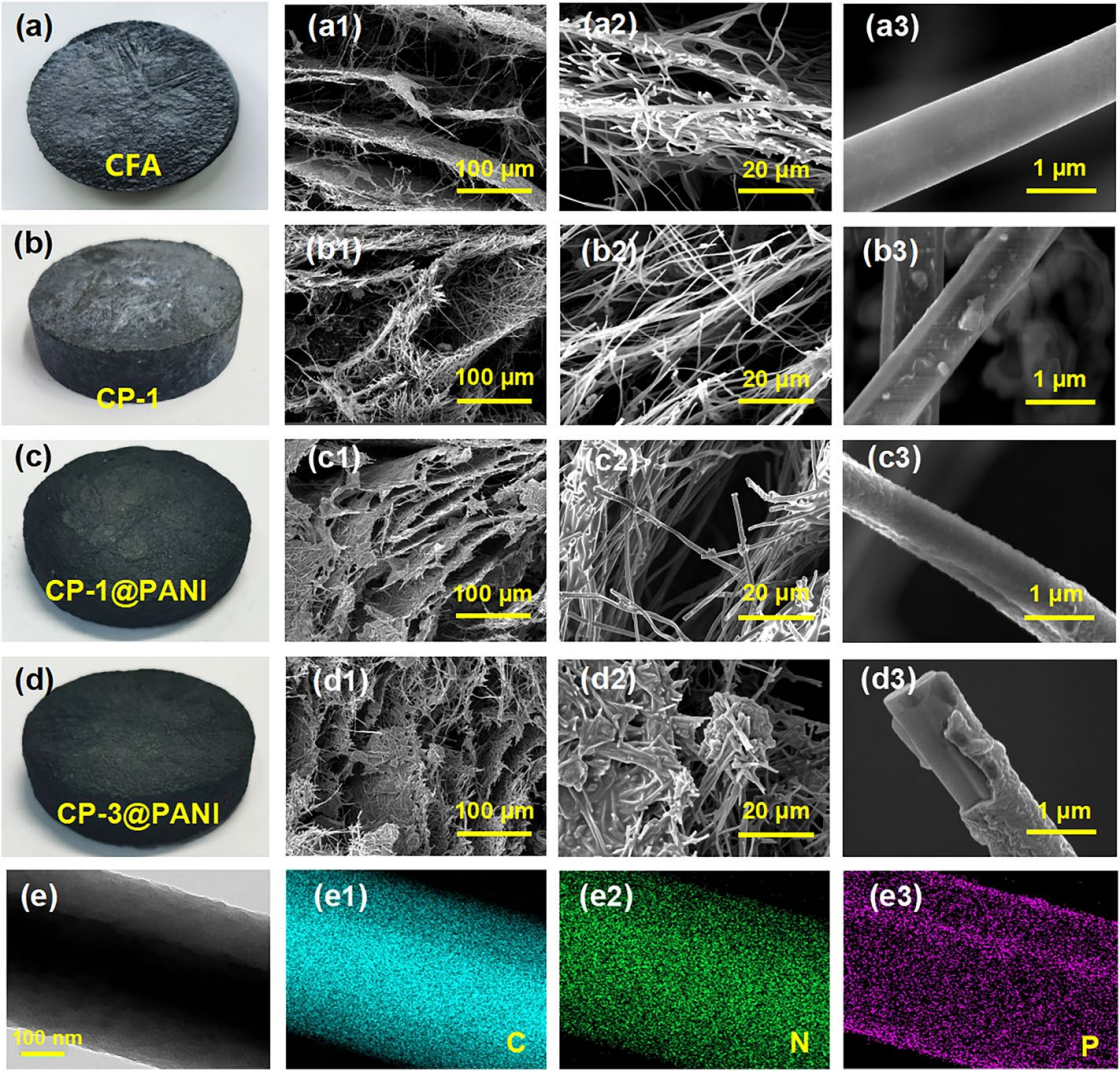
Digital images of **a** CFA—carbon nanofibers aerogel. **b** CP-1—aerogel mixed with carbon nanofibers and PANI at 1:1 mass ratio. **c** CP-1@PANI—PANI in situ grown on CP-1 aerogel **d** CP-3@PANI aerogel—PANI in situ grown on the CP-3 aerogel. SEM images at different magnifications: **a1–a2** CFA aerogel, **b1–b2** CP-1 aerogel, **c1–c2** CP-1@PANI aerogel, and **d1–d2** CP-3@PANI aerogel. **a3** CFA aerogel, showing smooth fiber surfaces. **b3** PANI particles attached to the surface of carbon nanofibers in the CP-1 aerogel. **c3** PANI uniformly covering the carbon nanofiber surface of the CP-1@PANI aerogel, and **d3** CP-3@PANI aerogel shows the PANI shell layer on the carbon nanofiber surface becomes more pronounced. **e** TEM images and **e1-e3** energy-dispersive X-ray spectroscopy (EDS) of CP-3@PANI

The cross-sectional SEM images show that the CFA has a fluffy structure with smooth fiber surfaces (Fig. 2a). Additionally, Figs. 2b and S9a, b reveal the presence of numerous PANI particles in the aerogel, with some of them attached to the surface of CNFs (Fig. 2b3). When CFA is immersed in an ANI solution, the fiber surfaces are covered with PANI (CFA@PANI, as depicted in Fig. S9c). When PANI particles introduced into CFA as the growing seed, after soaking in an ANI solution, PANI grows in situ along the CNFs, leading to the CNF being uniformly covered with PANI (CP-1@PANI, as illustrated in Fig. 2c). The above result can be rational as PANI in CFA forms hydrogen bonding with ANI, therefore attracting more ANI onto the aerogel for in situ polymerization. These hydrogen-bonded sites provide attachment points for in situ polymerization, forming the core–shell structure fibers (Fig. 2c1–d3). Notably, as the PANI content in CFA increases, the PANI shell layer on the fiber surfaces becomes more pronounced (Figs. 2d, S9d, and S10). The EDS results show that the CP-3@PANI aerogel contains the elements C, P, N, and O (Fig. S11). The TEM of the CP-3@PANI clearly depicts the core–shell morphology (Fig. 2e). To further validate the core–shell structure, we performed EDS mapping, which shows the distribution of nitrogen (N) and phosphorus (P) elements, confirming the presence of PANI on the surface of the CNFs (Fig. 2e1–e3).

The weight gain of the aerogels after polymerization also follows this trend (Table S2). After polymerization, CFA, CP-1, CP-2, and CP-3 show weight increases of 0.54, 0.63, 0.75, and 0.82 g, respectively, indicating a gradual increase in ANI consumption. This result stems from the hydrogen bonding interactions between PANI and ANI. As more ANI is attracted to the fiber surfaces, more PANI is generated after polymerization.

**Fig. 3:**
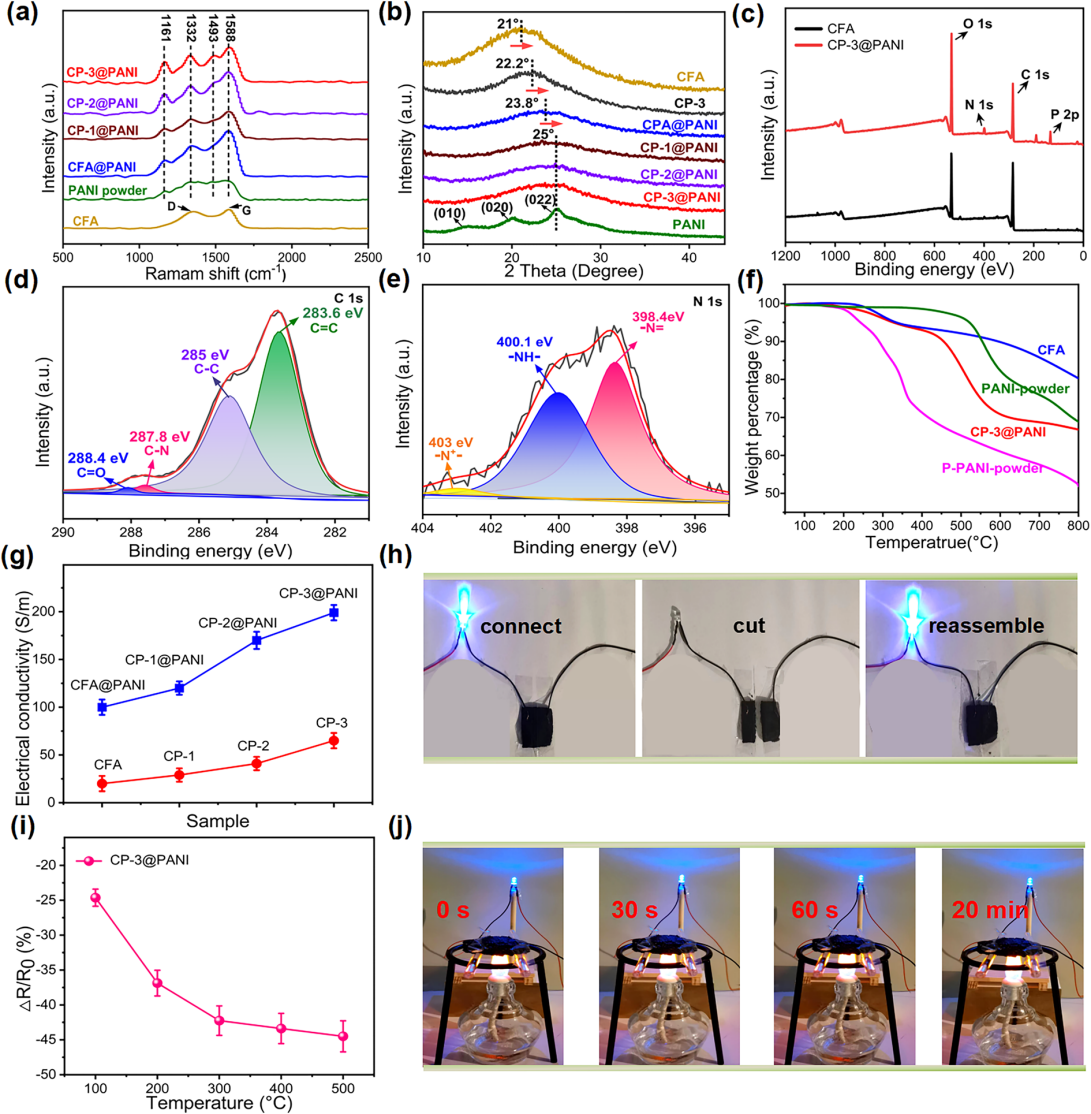
**a** Raman spectra of CFA, PANI powder, CFA@PANI, CP-1@PANI, CP-2@PANI, and CP-3@PANI. **b** XRD patterns of CFA, CP-3, CFA@PANI, CP-1@PANI, CP-2@PANI, and CP-3@PANI. **c** XPS spectra of CNFs and CP-3@PANI. **d** C 1*s* spectrum of CP-3@PANI. **e** N 1*s* spectrum of CP-3@PANI. **f** TGA curves of P-PANI powder, PANI powder, CNFs, and CP-3@PANI. **g** Conductivity of different aerogels. **h** Photograph of CP-3@PANI connected to an LED light. **i** Temperature-dependent resistance for CP-3@PANI. **j** Photograph of CP-3@PANI connected to an LED light under flame exposure

As shown in Figs. 3a and S12, the Raman spectrum of CFA displays two peaks at 1350 cm⁻^1^ (D band) and 1590 cm⁻^1^ (G band) in the first-order region, which are characteristic peaks of aromatic carbon [31]. After incorporating PANI into the aerogel, these two peaks broaden. The Raman spectrum of pure PANI exhibits peaks at 1588, 1493, 1332, and 1161 cm⁻^1^, corresponding to the stretching vibration of C–C in the benzene ring, the stretching vibration of C-N in the quinone ring, the stretching of C = N and the bending vibration of C-H in both benzene and quinone rings, respectively [32]. All four characteristic peaks of PANI appear in the Raman spectra of all hybrid aerogels, becoming more pronounced in the CFA@PANI, CP-1@PANI, CP-2@PANI, and CP-3@PANI. According to the XRD spectra (Fig. 3b), CFA shows a clear diffraction peak at 21°, corresponding to the (002) and (100) crystal planes in graphite microcrystals [33]. PANI powder has three diffraction peaks appearing at 15.1°, 20.4°, and 25°, and a strong peak at 25° (022) represents the d-spacing between benzene rings in parallel PANI chains [34]. As the PANI content increases, the 2-theta value of the core–shell structured aerogels shifts toward 25°, indicating an increased amount of PANI loaded onto the aerogel.

The XPS survey scan of the CP-3@PANI displays characteristic peaks of phytic acid (PA) and PANI, including P 2*p*, C 1*s*, N 1*s*, and O 1*s* elements (Fig. 3c). Figure 3d shows four energy bands centered at 283.6, 285.0, 287.8, and 288.4 eV in the C 1*s* spectrum, assigned to *sp*^2^-hybridized (C = C), *sp*^3^-hybridized (C–C), C–N, and carbonyl (C = O) groups, respectively [35, 36]. The fitted N1s spectrum of the phytic acid-doped PANI (denoted as P-PANI) (Fig. 3e) reveals three distinct peaks at approximately 398.5, 400.5, and 403.1 eV, attributed to quinoid imine (–N =), benzenoid amine (–NH–), and phytic acid-doped amine (–NH*–), respectively [37]. The chemical state of P 2*p* in the P-PANI sample can be fitted with two peaks at approximately 133.26 and 134.34 eV, corresponding to the 2*p*_3/2_ and 2*p*_1/2_ levels with an energy split of ΔΕ = 1.21 eV, indicating the presence of phosphate (PO_4_^3-^) groups (Fig. S13) [38].

The thermal stability of the aerogels was analyzed using TGA (Fig. 3f). The undoped PANI shows great thermal resistance with a decomposition temperature higher than 500 °C. Around 200–400 °C, P-PANI undergoes a series of degradation, where PA is hydrolyzed into inositol and orthophosphate (phosphoric acid) [39]. At 800 °C, the residual weight of P-PANI is 52.31 wt%. As for the carbon fiber aerogel (CFA), the excellent thermal resistance of the carbon nanofiber led to only two weight loss stages at 250–350 °C (approx. 7.0 wt%) and 600 °C, attributed to the decomposition of the nanocellulose binder. Upon incorporating PANI with CFA, the CP-1@PANI, CP-2@PANI, and CP-3@PANI exhibited commendable thermal stability, with the residual of 71, 68.47, and 67.1 wt% at 800 °C, respectively (Fig. S14). The slight decrease in residual weight corresponds to the increasing proportion of PANI.

The specific surface area and the pore structure of CFA and CP-3@PANI were evaluated using N_2_ adsorption–desorption isotherms. As shown in Fig. S15a, b, both samples exhibit type IV isotherms according to IUPAC, which is characteristic of mesoporous materials. The specific surface area and pore volume data are summarized in Table S3. The BET surface area and total pore volume of CP-3@PANI were significantly lower compared to CFA, due to the formation of a dense PANI shell on the surface of the CNFs during in situ polymerization. Furthermore, the pore size distribution, depicted in Fig. S15c, d, demonstrates that the pore sizes of both CFA and CP-3@PANI predominantly fall within the range of 2–50 nm, further confirming their mesoporous nature.

The CFA, CP-3, and CP-3@PANI all possess mesoporous structures facilitating swift water absorption. Additionally, they contain small amounts of nanocellulose, which possesses abundant hydroxyl (-OH) groups capable of forming hydrogen bonds with water molecules, contributing to the hydrophilicity of the aerogels. This is evidenced by the instantaneous water uptake observed in the contact angle tests for all samples (Fig. S16). In CP-3, PANI contains functional groups such as amines and imines, which enhance their hydrophilicity by forming hydrogen bonds with water molecules. In CP-3@PANI, where the PANI is doped with phytic acid, this doping process introduces additional ionic functional further improving its affinity for water.

### Electrical Conductivity of Aerogels

The electrical conductivity of the carbon nanofiber film is 180 S m^−1^, and the conductivity values of the aerogel samples are provided in Fig. 3g. All aerogels exhibit conductivities exceeding 1 S m⁻^1^, meeting the requirements for EMI shielding materials [40]. The conductivity of the aerogels increases with the PANI content. Figure 3g shows that the conductivity of the CFA, CP-1, CP-2, and CP-3 aerogels is 20, 32, 48, and 65 S m⁻^1^, respectively. This enhancement in conductivity is attributed to the incorporation of PANI into the aerogels, which creates conductive pathways. After the polymerization process, the conductive PANI was coated on the aerogel surface. As a result, the conductivity of CFA@PANI, CP-1@PANI, CP-2@PANI, and CP-3@PANI further increased from 100, 122, 175 to 199 S m⁻^1^. The higher conductivity of the core–shell structured aerogels is attributed to the denser conductive network formed by the in situ grown PANI on the aerogel substrate. This process not only coats the fibers but also interlinks them, resulting in the formation of a network of interconnected pores. In Fig. 3h, a light-emitting diode (LED) bulb connected in series with CP-3@PANI lights up when powered. When CP-3@PANI is disconnected, the circuit breaks and the LED bulb turns off. Reconnecting the two cross sections of CP-3@PANI causes the LED bulb to light up again at the same brightness, demonstrating that CP-3@PANI has good conductivity and a stable conductive network.

Figures 3i and S17 illustrate the temperature-dependent resistance of the CP-3@PANI. The resistance decreases with increasing temperature, indicating a negative temperature coefficient (NTC) effect in the CP-3@PANI, consistent with previous reports [41]. CP-3@PANI aerogel shows high conductivity over an alcohol lamp flame for 20 min, as indicated by the brightness of the LED light (Fig. 3j and Movie S1). We also study the hysteresis of NTC behavior of the CP-3@PANI. Figure S18a displays the temperature-dependent resistance undergoing heating and cooling cycles. Throughout multiple cycles, the resistance of CP-3@PANI consistently decreased during heating and recovered during cooling, demonstrating good reproducibility of the NTC behavior and flame resistance. Moreover, Fig. S18b shows the resistance of the CP-3@PANI dropped significantly (~ 17.5% ΔR/R_0_) within ~ 1 s, indicating its potential application as the first responder for fire alarm systems.

### EMI Shielding Performance of Aerogels

**Fig. 4:**
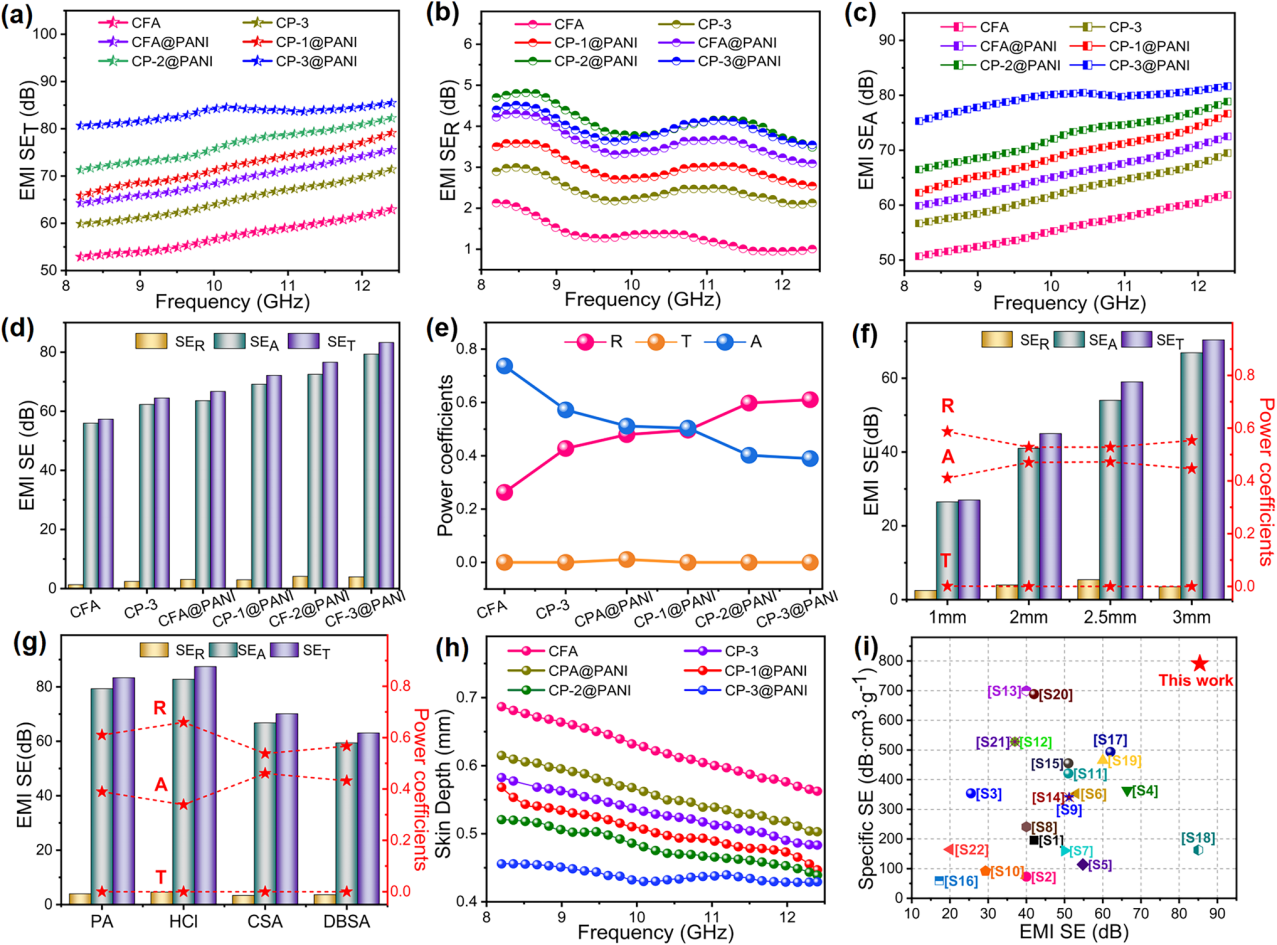
**a** SE_T_ values, **b** SE_R_ values, **c** SE_A_ values, **d** average SE_T_, SE_R_, and SE_A_ values, and **e** R, T, and A values of CFA, CP-3, CFA@PANI, CP-1@PANI, CP-2@PANI, CP-3@PANI in the X-band. **f** EMI shielding performance of CP-3@PANI with different thicknesses. **g** EMI shielding performance of CP-3@PANI with different acid doping. **h** Skin depth of CFA, CP-3, CFA@PANI, CP-1@PANI, CP-2@PANI, CP-3@PANI. **i** Comparison of SE d^−1^ values from this work with related EMI aerogels results

The materials' conductivity determines their effectiveness in electromagnetic shielding, showing the potential of these aerogels as high-performance EMI shielding materials. The EMI SE of the aerogels is shown in Fig. 4a, with measurements conducted in the 8.2 − 12.4 GHz frequency range (X-band). CNFs exhibit EMI SE of 62.9 dB. Upon the addition of PANI, the SE_T_ value of the aerogels increases to 71.3 dB. The SE_T_ values of aerogels based on core–shell CFA@PANI are even more remarkable as the SE_T_ value increases to 75.4 dB presumably due to the synergy between CNFs and PANI. As the PANI content increases, the SE_T_ values of CP-1@PANI, CP-2@PANI, and CP-3@PANI show a significant upward trend, increasing from 79 to 85.4 dB. This is due to the hydrogen bonding effect, where PANI attracts more ANI to polymerize on the aerogels, forming a high conductivity and interconnected 3D conductive network, effectively dissipating electromagnetic energy [42]. An increase in PANI content results in the formation of a more extensive and well-connected conductive network, and leading to more mobile charge carriers, which contribute to the enhancement of SE_R_ (Fig. 4b). The strong interfacial polarization generated by the numerous interfaces between the PANI shell and the carbon nanofiber core facilitates an increase in SE_A_ (Fig. 4c). Furthermore, the abundant pores in the aerogel enhance multiple reflections and scatterings of incident EMW, intensifying EM waves-material interactions and leading to a steady increase in both SE_A_ and SE_T_ with higher PANI content [43]. The average values of SE_T_, SE_A_, and SE_R_ for the aerogels also reflect this trend (Fig. 4d). The SE of CP-3@PANI is 85.4 dB, exceeding the SE of pure CFA by 35.8% and far surpassing the 20 dB required for commercial EMI shielding materials.

The power coefficients quantify the ability of material to absorb, reflect, and transmit EMW, including the transmission coefficient (T), reflection coefficient (R), and absorption coefficient (A). As shown in Fig. 4e, the A value of CFA is much greater than the R value. For CP-3, the A value decreases to 0.57 but remains greater than the R value, indicating that EMI shielding is primarily based on the absorption mechanism. Studies have shown that CNFs can create scattering and multiple internal reflection effects. This lengthens the propagation distance of incident EMW and enhances electromagnetic wave absorption loss through destructive interference [44]. Additionally, the 3D porous structure improves impedance matching at the material–air interface, thus enhancing electromagnetic wave absorption [45]. As the PANI content increases, the R value of core–shell aerogels gradually exceeds the A value, suggesting a shift from an absorption-dominated to a reflection-dominated EMI shielding mechanism. This is due to the increase in material conductivity, causing an impedance mismatch at the material–air interface, resulting in partial reflection of EMW on the material surface [46]. Additionally, the EMI shielding performance is closely linked to the material's thickness. Thicker aerogels generally have higher EMI SE values. As depicted in Fig. 4f, the 1-mm-thick CP-3@PANI exhibits an EMI SE of 27 dB. As the aerogel thickness increases, the EMI SE significantly improves, attenuating 99.99% of incident EMW at a thickness of 3 mm. Doping of PANI with different protonic acids also has a huge impact on the EMI shielding performance. Figure 4g shows that doping of CP-3@PANI with HCl results in the highest EMI SE, followed by PA, CSA, and DBSA. This trend aligns with the conductivity of PANI in different doping states (Fig. S19). HCl is a strong acid with high ionization strength, providing more H^+^ ions to bond with N in the quinonoid structure of PANI, reducing the quinone ring to a benzene ring. The more charge carriers in HCl doped PANI leads to the highest conductivity. DBSA, being the weak acid and the largest anion size with poor mobility, results in the lowest conductivity for doped PANI [47]. Adjustment of the reaction time was adopted to control the shell thickness, and we conducted in situ polymerization experiments on CP-3 aerogels for 4, 8, 12, and 16 h. The PANI shell thickens progressively with increasing reaction time (Fig. S20). Correspondingly, the EMI SE at 12.4 GHz improved from 78.2 to 82.6, 84.5, and 84.6 dB, respectively (Fig. S21). However, after 12 h, the growth in EMI SE slowed down, with the 16-h polymerization providing only a slight improvement over the 12-h sample. This phenomenon could be attributed to the saturation of the PANI layer or the reduced effectiveness of additional material for enhancing EMI SE beyond a certain thickness.

We also investigated the skin depth (δ), an important parameter for evaluating EMI shielding capability, as defined in formula (S10). Notably, the skin depth refers to the depth at which the intensity of the incident EMW attenuates to 1/e of its original value as it penetrates the shielding material. As shown in Fig. 4h, the δ values of all aerogels are lower than their thickness (~ 4.5 mm). Compared to CFA, the δ values of aerogels decrease after adding PANI, with the δ value of CP-3@PANI decreasing to 0.46 mm, indicate excellent EMI shielding performance. To better evaluate the performance of EMI shielding materials, we compared the SSE d⁻^1^ values of CP-3@PANI with other reported carbon-based and PANI-based aerogel EMI shielding materials (Fig. 4i, Table S4). The specific SE value of the CP-3@PANI is as high as 791.2 dB cm^3^ g⁻^1^, surpassing the performance of most similar EMI shielding materials.

**Fig. 5:**
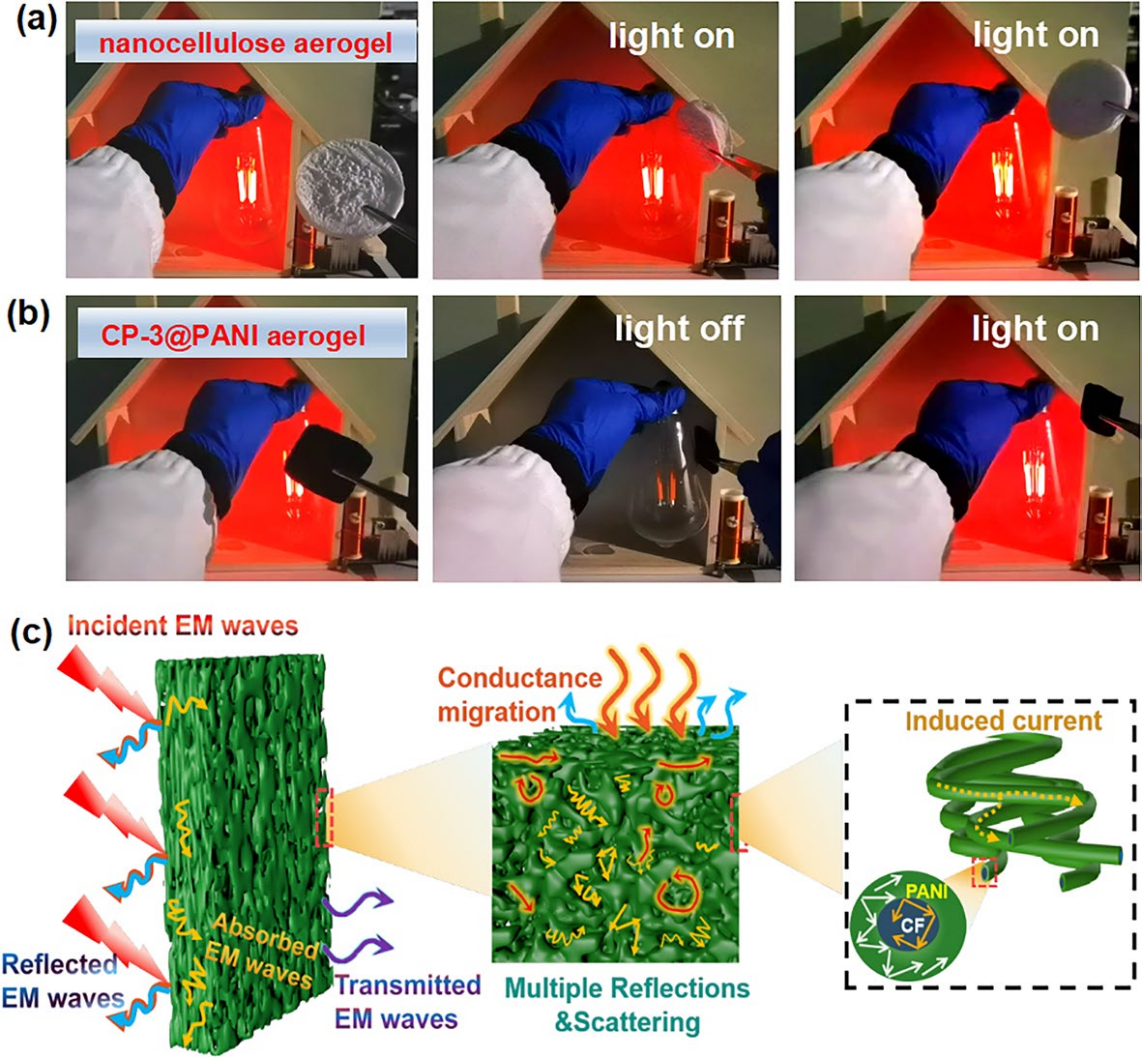
**a** Photograph of electromagnetic shielding by the pure nanocellulose aerogel, showing that the EMW signal has not been effectively shielded. **b** Photograph of electromagnetic shielding by CP-3@PANI, demonstrating that the EMW signal is effectively shielded. **c** EMI shielding mechanism of CP-3@PANI including the incident, reflected, absorbed, and transmitted electromagnetic fields

To demonstrate the commercial implications of CP-3@PANI as an EMI shielding material, we utilized a Tesla coil wireless power transmission system operating at a frequency of 6.356 MHz as the EM emission source [48]. The light bulb could be illuminated through a wooden board when the Tesla coil was activated. When pure nanocellulose aerogel was placed between the Tesla coil and the incandescent bulb, the brightness of the bulb remained unchanged (Fig. 5a). However, when CP-3@PANI was placed between the Tesla coil and the incandescent bulb, the bulb immediately turned off, and it relit as soon as CP-3@PANI was removed (Fig. 5b and Movie S2). This phenomenon demonstrates that CP-3@PANI effectively shielded the EMW signal, providing EMI shielding with a performance of 10.27 dB at 6.356 MHz (Fig. S22). To provide a more comprehensive evaluation, we have expanded our testing to include the low-frequency range from 50 to 500 MHz, as well as the K-band. The average shielding effectiveness in the 50 to 500 MHz range is 15.6 dB (Fig. S23), while in the K-band, it reaches 94.4 dB. This broad-spectrum analysis highlights that our material exhibits strong performance not only in the X-band but also in lower-frequency bands and the K-band, demonstrating its suitability for protection in a wide range of electronic products (Fig. S24).

The permittivity of the materials was measured to analyze their intrinsic dielectric properties. As shown in Fig. S25, both CFA and CP-3@PANI exhibit frequency-dependent behavior in their real (ε') and imaginary (ε'') permittivity. In the 8.2–12.4 GHz frequency range, both ε' and ε'' values decrease, characteristic of dielectric dispersion [49]. For CFA, the ε' value decreases from 7.4 to 6.7. In contrast, the ε' value of CP-3@PANI fluctuates between 9.8 and 8.2, indicating a stronger capacity for electric energy storage. Similarly, CP-3@PANI exhibits the highest ε'' values, ranging from 15.1 to 11.2, reflecting its superior ability to dissipate electrical energy from incident electromagnetic waves. The higher ε' and ε'' values of CP-3@PANI compared to CFA indicate enhanced dielectric storage and loss capabilities, contributing to its superior performance in electromagnetic energy attenuation [50]. Figure 5c illustrates the electromagnetic shielding mechanism of CP-3@PANI. The aerogel's CNFs are coated with a layer of PANI, which has free electron effects, causing direct reflection of some of the EMW. A significant impedance mismatch exists between the air and the highly conductive material interface [51]. As a result, some of the incident EMW are immediately reflected upon reaching the material's surface, while the rest are absorbed inside the aerogel [52]. The EMI shielding properties of the material can be explained by three main factors: dipole polarization, interfacial polarization, and conductive network pathways (conductivity). First, the conductive network provided by PANI greatly contributes to electron hopping, significantly enhancing EMI shielding performance [53]. Second, interfacial polarization at the heterogeneous interface between the PANI shell and the CNF core further dissipates EMW energy. Additionally, the numerous polarons and dipoles on PANI also contribute to EMW attenuation [54]. Furthermore, the mesoporous structure within the skeletal network of aerogel enables multiple reflections and scatterings of incident EMW, converting more electromagnetic energy into heat or other forms of energy for absorption or dissipation [55, 56]. All the above features in the CP-3@PANI lead to high EMI shielding performance.

### Thermal Insulation Performance of Aerogels

**Fig. 6:**
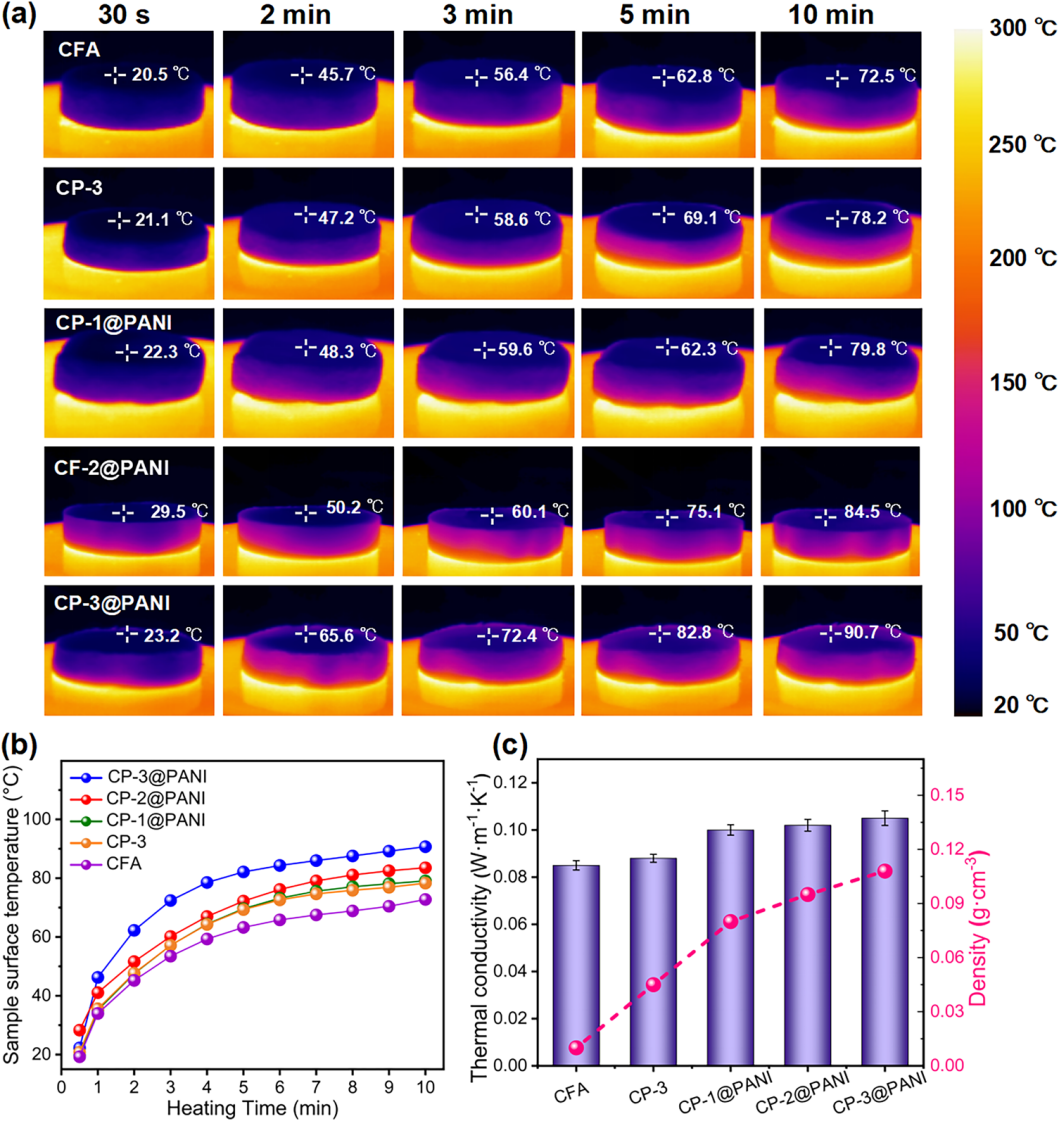
**a** Thermal infrared images of CFA, CP-3, CFA@PANI, CP-1@PANI, CP-2@PANI, and CP-3@PANI, showing the temperature on the top surface of these aerogels when placed on a 300 °C heating platform for 0–10 min. **b** Time-dependent surface temperature corresponding to **a**.**c** Thermal conductivity and density of these aerogels, showing they are effective insulation materials

Thermal stability is an important factor when evaluating electromagnetic shielding materials, as it helps to maintain optimal performance in high temperatures and prevents thermal damage. We placed samples approximately 10.6 mm thick on a heating plate set to 300 °C, with the bottom surface in direct contact with the plate, and captured thermal infrared images of the samples at 30 s and 2, 3, 5, 10 min (Fig. 6a). After 10 min of continuous heating, the surface temperature of the CFA was 72.5 °C. With increasing PANI content, the surface temperature of the samples slightly increased, with the CP-3@PANI surface temperature reaching 90.7 °C, demonstrating good thermal insulation performance (Fig. 6b). In essence, all samples exhibit good thermal insulation. At 20 °C and 45% relative humidity, we measured CFA's room temperature thermal conductivity. The material has a very low thermal conductivity of about 0.085 W m⁻^1^ K⁻^1^ due to its high porosity (82.2%), in which air acts as a barrier to heat transfer and convection [57]. The addition of PANI did not significantly change the thermal conductivity of the aerogels. The core–shell aerogels have thermal conductivities exceeding 0.1 W m⁻^1^ K⁻^1^. In the case of CP-3@PANI, the thermal conductivity was measured at 0.104 W m⁻^1^ K⁻^1^ (Fig. 6c). As the PANI content increases, its inherent conductive properties contribute to a higher thermal conductivity [58]. The process of incorporating PANI also causes slight structural changes within the aerogel. After the in situ polymerization and impregnation of PANI, the aerogel undergoes a degree of shrinkage, leading to an increase in density and a decrease in porosity. This reduction in porosity diminishes the aerogel's ability to trap air and hinder heat transfer through convection. Consequently, the more compact structure with lower porosity allows for more efficient heat conduction, thus contributing to the rise in thermal conductivity. Even so, the overall thermal conductivity remains in a low level, preserving the material’s effective thermal insulation properties. To further evaluate the thermal stability of CP-3@PANI, we tested its thermal insulation performance, thermal conductivity, and EMI shielding effectiveness (EMI SE) after 10 repeated heating and cooling cycles between room temperature (RT) and 300 °C. As shown in Figs. S26–S28, CP-3@PANI exhibited only minor changes after thermal cycling. These results demonstrate the robust thermal stability of CP-3@PANI, confirming its suitability as a fire-retardant EMI shielding material for applications in thermally fluctuating environments.

**Fig. 7:**
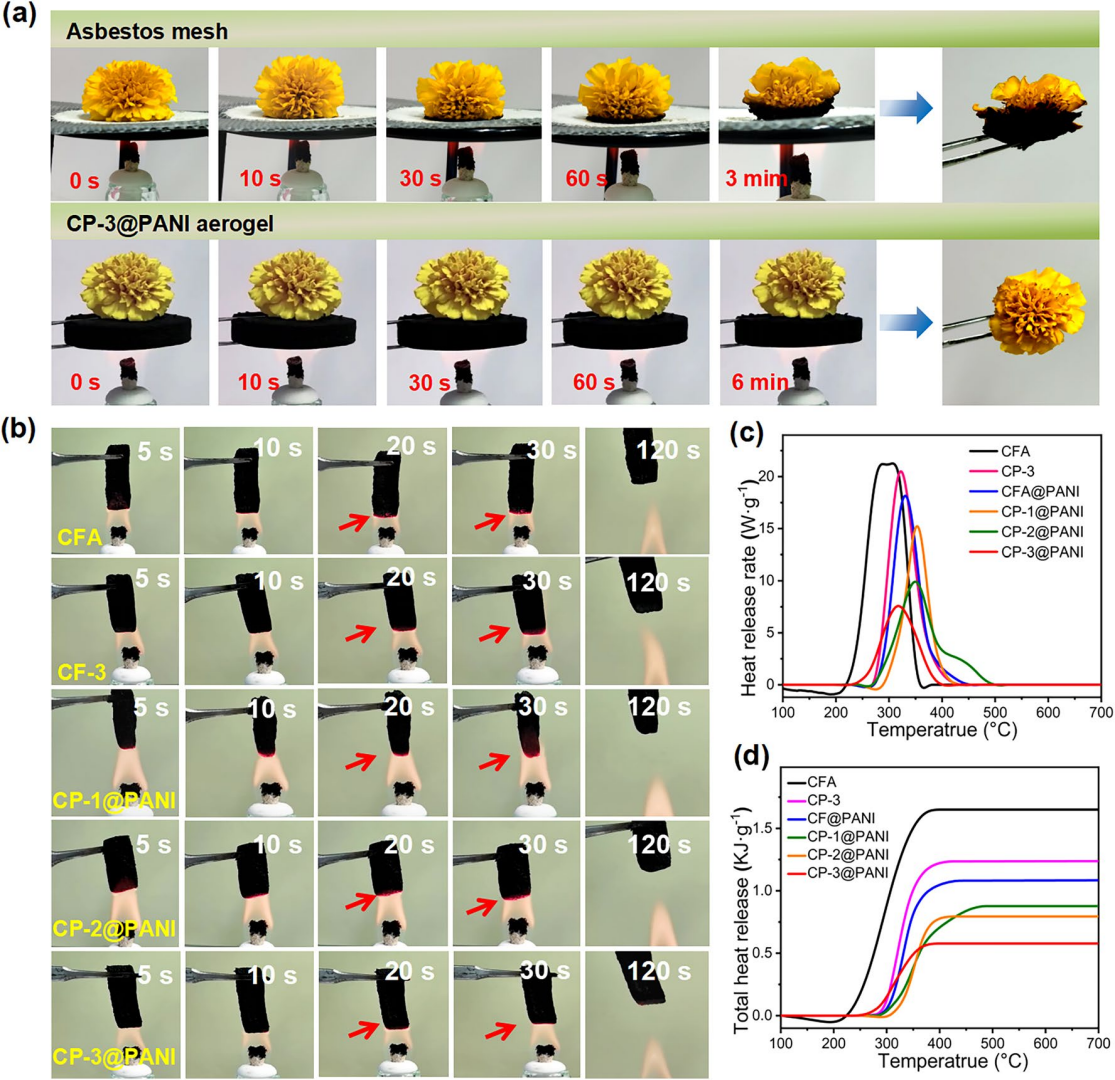
**a** Comparison of thermal insulation performance between asbestos mesh and CP-3@PANI. It shows that the flower is unaffected, and CP-3@PANI remained intact even after 6 min of burning. **b** Digital photos of actual burning of CFA, CP-3, CFA@PANI, CP-1@PANI, CP-2@PANI, and CP-3@PANI, showing the reduced glowing area of the CP-3@PANI in the flame. **c** HHR curves. **d** THR curves of these aerogels. CP-3@PANI shows low values of 7.9 W g⁻^1^ and 0.58 kJ g⁻^1^

To visually demonstrate the thermal insulation performance of the material, we placed a flower on asbestos mesh and CP-3@PANI, respectively, and heated them with an alcohol lamp for comparison (Fig. 7a). The flower on the asbestos mesh began to wilt within just 1 min and was severely damaged at the bottom after 3 min. In contrast, the flowers on top of the CP-3@PANI remained intact even after 6 min of burning. This clearly indicates that CP-3@PANI is an effective thermal insulation material.

The thermal insulation performance of the CP-3@PANI is primarily attributed to the effective suppression of heat conduction, convection, and radiation. The porous structure, filled with air that has low thermal conductivity, significantly reduces solid contact and slows down heat conduction [59]. Additionally, the complex pore network limits air movement, thereby minimizing thermal convection. The large specific surface area of the aerogel further diminishes heat radiation through reflection and scattering [60]. Overall, the CP-3@PANI demonstrates excellent thermal insulation due to its multifaceted mechanisms of inhibiting heat transfer, ensuring protection from thermal damage in high-temperature environments.

### Thermal Stability and Combustion Behavior of Aerogels

Phytic acid-doped polyaniline (P-PANI) is an excellent flame-retardant material because its high phosphorus content can provide a more active flame-retardant element, with PA containing up to 28 wt% phosphorus (based on molecular weight) [39]. The PA content in doped PANI, along with the calculation method, is provided in Table S2 and Fig. S29. PANI, which has a high carbon residue due to the formation of the graphitic structure during pyrolysis, creates a strong barrier that prevents the transmission of oxygen, heat, and flammable substances. Meanwhile, pyrolysis releases non-flammable NH_3_, N_2_, and H_2_O vapor, further enhancing the aerogels' flame-retardant properties. Digital photos in Fig. 7b depict the burning process of the samples under continuous exposure to an alcohol lamp flame for 120 s. Although CFA glows during this period, it does not ignite. This can be attributed to the excellent heat resistance of the CNFs, which maintain the material's structural integrity and prevent combustion. High porous aerogel also facilitates rapid heat dissipation and prevents combustion. Notably, the glowing area of the CP-3@PANI in the flame is reduced (Movie S3). To further assess the heat release behavior, a microscale combustion calorimeter (MCC) was utilized, and the heat release rate (HRR) as well as total heat release (THR) curves are depicted in Fig. 7c, d, with corresponding data provided in Table 1. The HRR curve of CFA shows a broad peak at 200–385 °C, with a Peak heat release rate (PHRR) value of 21.9 W g⁻^1^ at 311.9 °C, attributed to the presence of flammable nanocellulose. Considering that pure nanocellulose aerogel has a PHRR of 248.9 W g⁻^1^ at 322.7 °C (Fig. S30, Table S5), it suggests that the heat generated by CFA combustion at 700 °C mainly results from nanocellulose. More importantly, after adding PANI, the PHRR of the aerogels further decreases. The CP-3@PANI, with the highest PANI content, shows the most significant decrease in PHRR, down to only 7.8 W g⁻^1^. THR is another parameter used to assess fire hazard, calculated from the area under the HRR curve. For CFA, the THR is 1.65 kJ g⁻^1^. As the PANI content increases, the samples' THR and heat release capacity (HRC) gradually decrease. The THR of CP-3@PANI is only 0.58 kJ g⁻^1^, approximately 64.8% lower than CFA's. This indicates excellent fire safety performance with extremely low PHRR and THR values. Furthermore, the flame retardancy of the composite aerogels was evaluated using the LOI test (Fig. S31). The pure nanocellulose aerogel exhibited an LOI value of 17.1%, reflecting its low flame resistance. In contrast, the CFA demonstrated a significant increase in LOI to 42%, which can be attributed to the high thermal stability of the CNFs. Upon the addition of PANI to CFA, the LOI value of CP-3 further increased to 55%, indicating the synergistic effect of PANI in enhancing the material's flame-retardant properties. Most notably, after the in situ polymerization of PANI with PA, the LOI value of CP-3@PANI reached 68%. This remarkable enhancement is due to the high phosphorus content in PA, which promotes the formation of a phosphate-rich protective layer during combustion. This layer effectively limits the exposure of the material to oxygen, suppresses oxidation, and improves fire safety.

**Table 1:** Related MCC Data of CFA and composite aerogels

Sample	PHRR (W g^−1^)	T*p* (°C)	THR (kJ g^−1^)	HRC (J g^−1^ K^−1^)
CFA	21.9	311.9	1.65	21.7
CP-3	20.5	323.1	1.23	21.0
CFA@PANI	18.3	332.2	1.06	20.5
CP-1@PANI	15.9	353.7	0.78	15.8
CP-2@PANI	10.9	349.2	0.87	10.8
CP-3@PANI	7.8	310.2	0.6	7.7

**Fig. 8:**
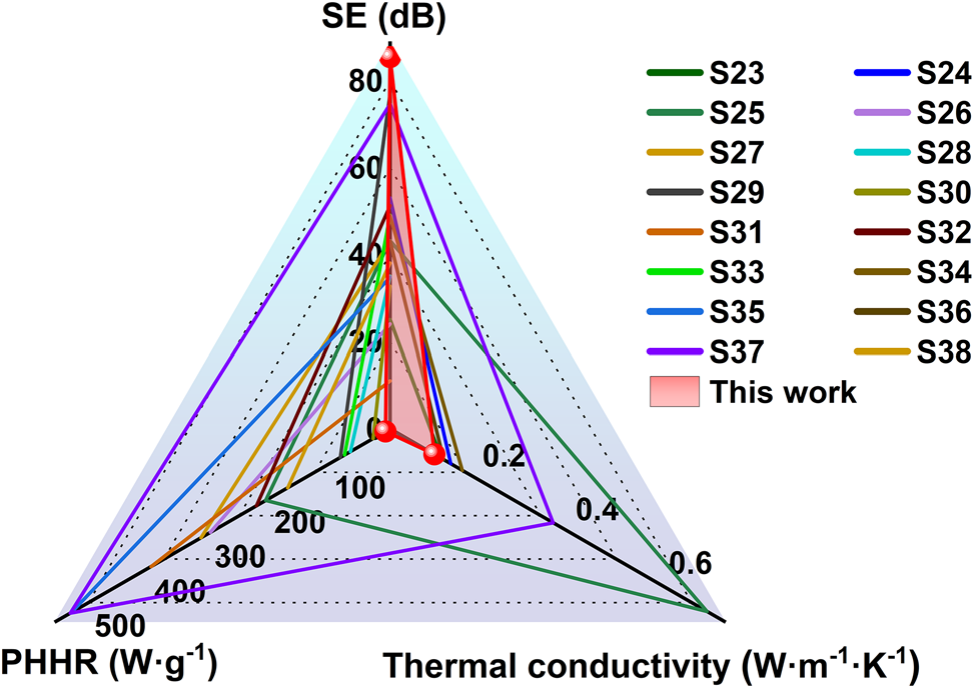
Comprehensive performance comparison between CP-3@PANI and samples reported in the literature

To put things in perspective, we have summarized the results and compared CP-3@PANI with other multifunctional integrated materials, as shown in Fig. 8 and Table S6, the samples prepared in this work exhibit superior EMI shielding performance, extremely low peak heat release value, and low thermal conductivity. As can be seen in Fig. 8, our EMI shielding material integrating CNF core and PANI shell has performance characteristics that stand out among recent reported literature.

## Conclusion

In summary, we have successfully constructed a multifunctional aerogel composite consisting of a carbon fiber core and a PANI shell layer with excellent EMI shielding, thermal insulation, and flame retardancy properties. The superior structural design endowed the CP-3@PANI aerogel with high electrical conductivity (199 S m⁻^1^) and an excellent EMI SE (85.4 dB). Additionally, we ingeniously doped PANI with phytic acid, which has high flame retardancy, achieving ternary synergistic flame retardancy. The PHRR and THR of CP-3@PANI were as low as 7.9 W g⁻^1^ and 0.58 kJ g⁻^1^, respectively, reducing by 65.8% and 63.6% compared to CFA, demonstrating a fire barrier capability. Furthermore, the three-dimensional porous structure and component synergy endowed CP-3@PANI with excellent thermal insulation performance. The material exhibited an NTC effect in fire or high-temperature environments, maintaining its conductivity. Therefore, the CP-3@PANI has significant potential for application in building protective layers in the military, electronics, and industrial fields.

## Supplementary Information

Below is the link to the electronic supplementary material.

Supplementary file1 (DOCX 8256 kb)

Supplementary file2 (MP4 101304 kb)

Supplementary file3 (MP4 76363 kb)

Supplementary file4 (MP4 65606 kb)
