# Supplementary material for: Carbon Nanofiber/Polyaniline Composite Aerogel with Excellent Electromagnetic Interference Shielding, Low Thermal Conductivity, and Extremely Low Heat Release
Source: Nanomicro Lett. 2024 Dec 2;17:80. doi: 10.1007/s40820-024-01583-2 (PMC11609142; doi:10.1007/s40820-024-01583-2)
Supplement: Supplementary file 1 — Supplementary file1 (DOCX 8256 kb) [file 40820_2024_1583_MOESM1_ESM.docx]

Supporting Information for

**Carbon Nanofiber/Polyaniline Composite Aerogel with Excellent Electromagnetic Interference Shielding, Low Thermal Conductivity, and Extremely Low Heat Release**

Mingyi Chen^1,2†^, Jian Zhu^2†^, Kai Zhang^2^, Hongkang Zhou^2^, Yufei Gao^2^, Jie Fan^1,5^*, Rouxi Chen^2,3^*, Hsing-Lin Wang^2,3,4^*

^1^School of Textiles Science and Engineering, Tiangong University, Tianjin 300387, P. R. China

^2^Department of Materials Science and Engineering, Southern University of Science and Technology, Shenzhen, Guangdong 518055, P. R. China

^3^School of Innovation and Entrepreneurship, Southern University of Science and Technology, Shenzhen 518055, P. R. China

^4^Guangdong Provincial Key Laboratory of Energy Materials for Electric Power, Southern University of Science and Technology, Shenzhen, Guangdong 518055, P. R. China

^5^Ministry of Education Key Laboratory of Advanced Textile Composite Materials, Tiangong University, Tianjin 300387, P. R. China

†Mingyi Chen and Jian Zhu contributed equally to this work.

* Corresponding authors. E-mail: [fanjie@tiangong.edu.cn](mailto:fanjie@tiangong.edu.cn) (Jie Fan); [chenrx@sustech.edu.cn (Rou](mailto:chenrx@sustech.edu.cn%20(Rou)xi Chen); [wangxl3@sustech.edu.cn](mailto:wangxl3@sustech.edu.cn)(Hsing-Lin Wang)

**S1 Calculation**

EMI SE was measured using an Agilent E5071C vector network analyzer by the wave guide method in the 8.2-12.4 GHz (X-band) range. The measured scattering parameters (Figs. S11 and S21) were used to calculate the absorption, reflection, and total shielding values. EMI SE (SE_T_) is divided into three parts: reflection loss (SE_R_), absorption loss (SE_A_), and multiple reflection loss (SE_M_), and can be expressed by the following formulas:

$EMI SE=10lg\frac{P_{0}}{P_{1}}={SE}_{R}+{SE}_{A}+{SE}_{M}$ (S1)

When $\mathrm{SE}_{T}$≥15 dB，$\mathrm{SE}_{M}$ can be neglected, meanwhile，$\mathrm{SE}_{T}$、$\mathrm{SE}_{R}$and $\mathrm{SE}_{A}$ can be determined as follows：

${SE}_{T}={SE}_{A}$+${SE}_{R}$ (S2)

The power coefficients of reflection (R), transmission (T), and absorption (A) can be calculated based on the four scattering parameters (Figs. S11, S12, S21, and S22) of the network analyzer.

$R=\left| S_{11} \right|$*^2^ =* $\left| S_{22} \right|$*^2^* （S3）

$T=\left| S_{12} \right|$*^2^ =* $\left| S_{21} \right|$*^2^* （S4）

$A=1-R-T （S5）$

$\mathrm{SE}_{A}$ and $\mathrm{SE}_{R}$can be obtained as：

${SE}_{R}=10log\left( \frac{1}{1-R} \right)=10log\left( \frac{1}{1-\left| S_{11} \right|^{2}} \right)$（S6）

${SE}_{A}=10log\left( \frac{1-R}{T} \right)=10log\left( \frac{1-\left| S_{11} \right|^{2}}{\left| S_{21} \right|^{2}} \right)$（S7）

The skin depth (δ), defined as the depth at which the incident electromagnetic wave decays to 1/e of its surface value, can be calculated using the following formula (Gupta, Singh, Teotia et al., 2013):

$\delta=1/\sqrt{\pi f\mu\sigma}$ （S8）

where $f$ is the frequency, μ is the permeability, and σ is the conductivity.

Hence

$\mathrm{SE}_{A}=8.68 d\sqrt{\pi f\mu\sigma}$ (S9)

$\delta$ can also be obtained by the following formula：

$\delta=8$.68 （$d/{{SE}_{A}}$） (S10)

The specific SE (SE·d^-1^), which considers the material's density, is expressed as:

$\frac{SE}{d}=\frac{SE}{density}$ $(dB▪{cm}^{3}\cdot g^{-1})$(S11)

**S2 Supplementary Figures and Tables**


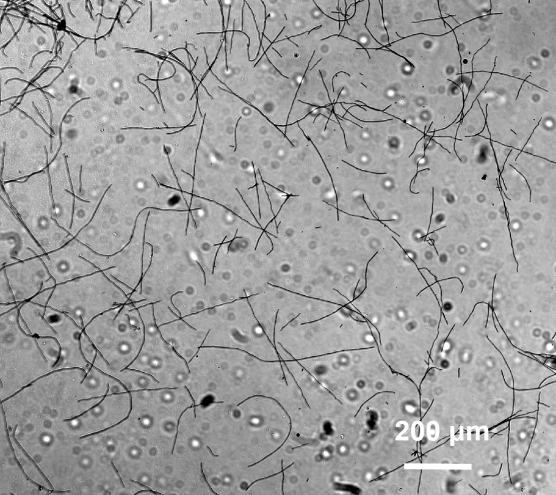


**Fig. S1** Microscopic image of the CNFs after grinding


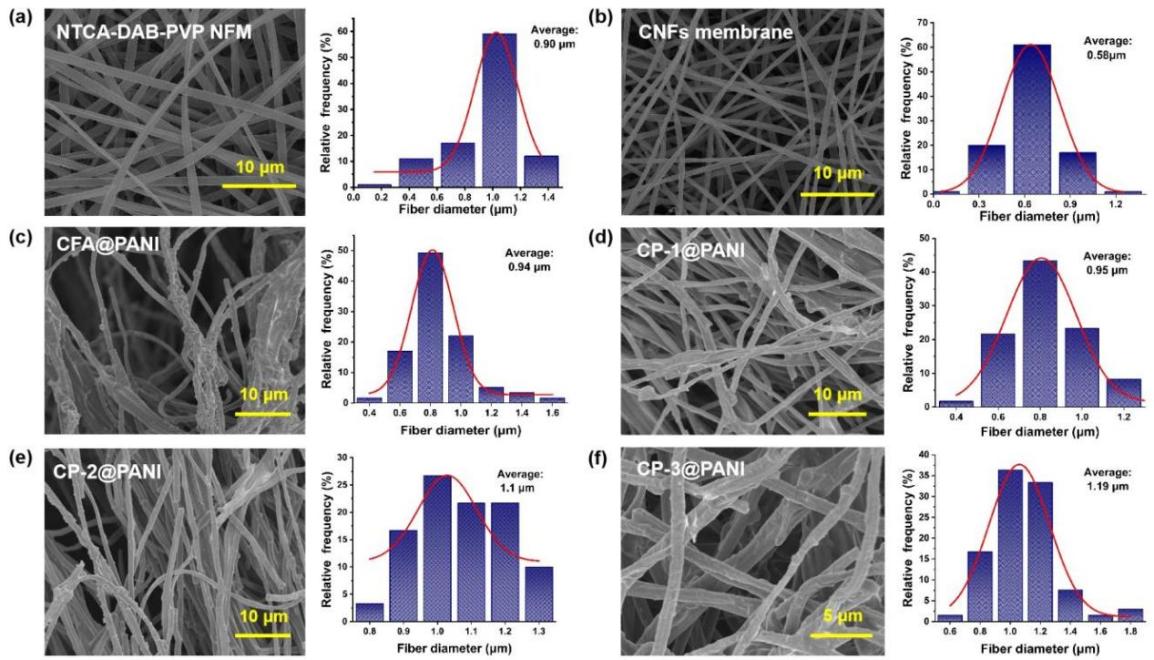


**Fig. S2** SEM images and diameter distribution diagrams of **a** NTCA-DAB-PVP nanofiber membrane. **b** carbon nanofibers membrane. **c** CFA@PANI. **d** CP-1@PANI. **e** CP-2@PANI. f CP-3@PANI


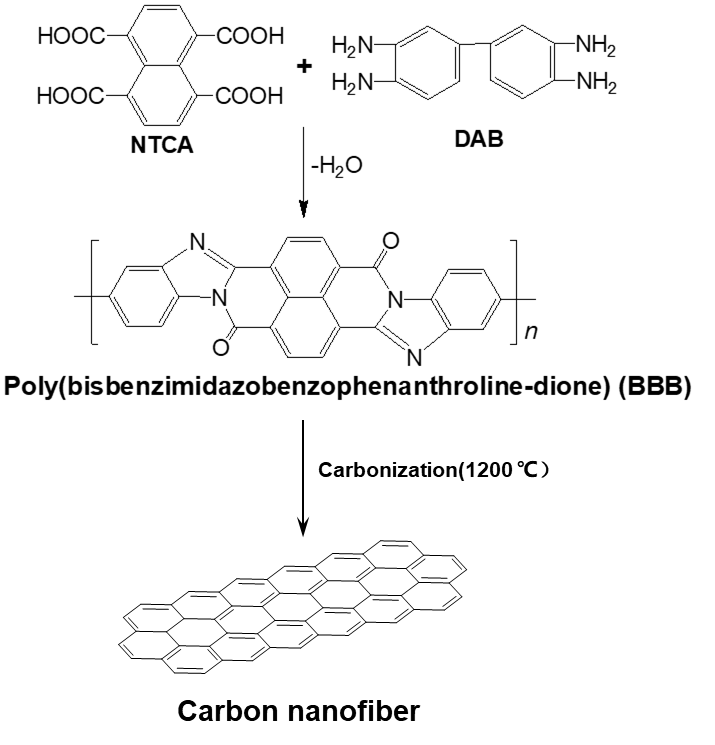


**Fig. S3** Synthetic scheme for the reaction of the NTCA and DAB monomers to BBB and derived carbon structure


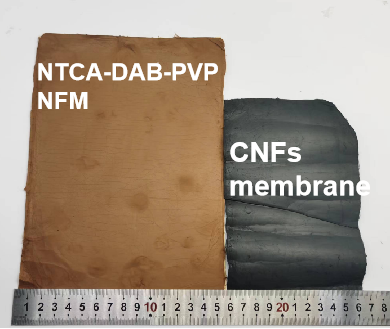


**Fig. S4** Comparative photos of NTCA-DAB-PVP membrane and its nanofiber membrane


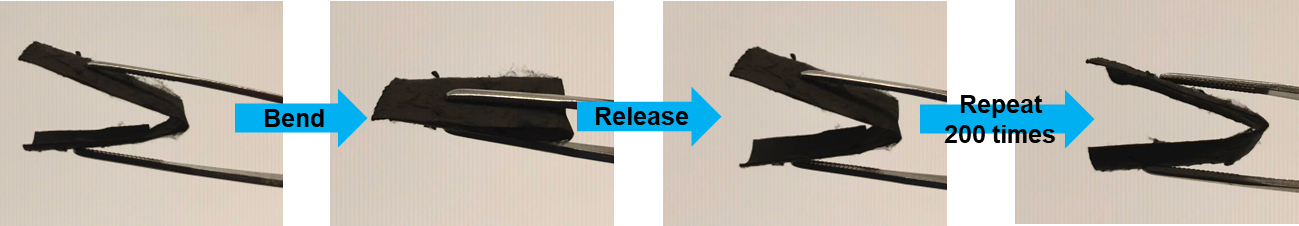


**Fig. S5** Photos of mechanical flexibility of CNFs membrane


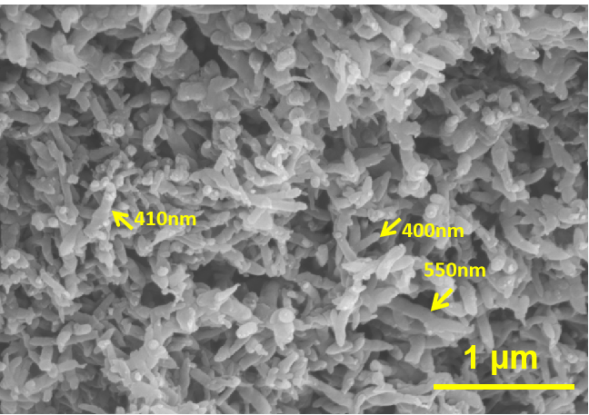


**Fig. S6** SEM image of PANI nanoparticles


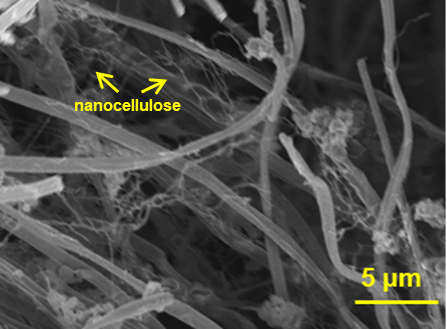


**Fig. S7** SEM image of in CFA aerogel, where the nanocellulose entangled around the CNFs and act as the binder

**
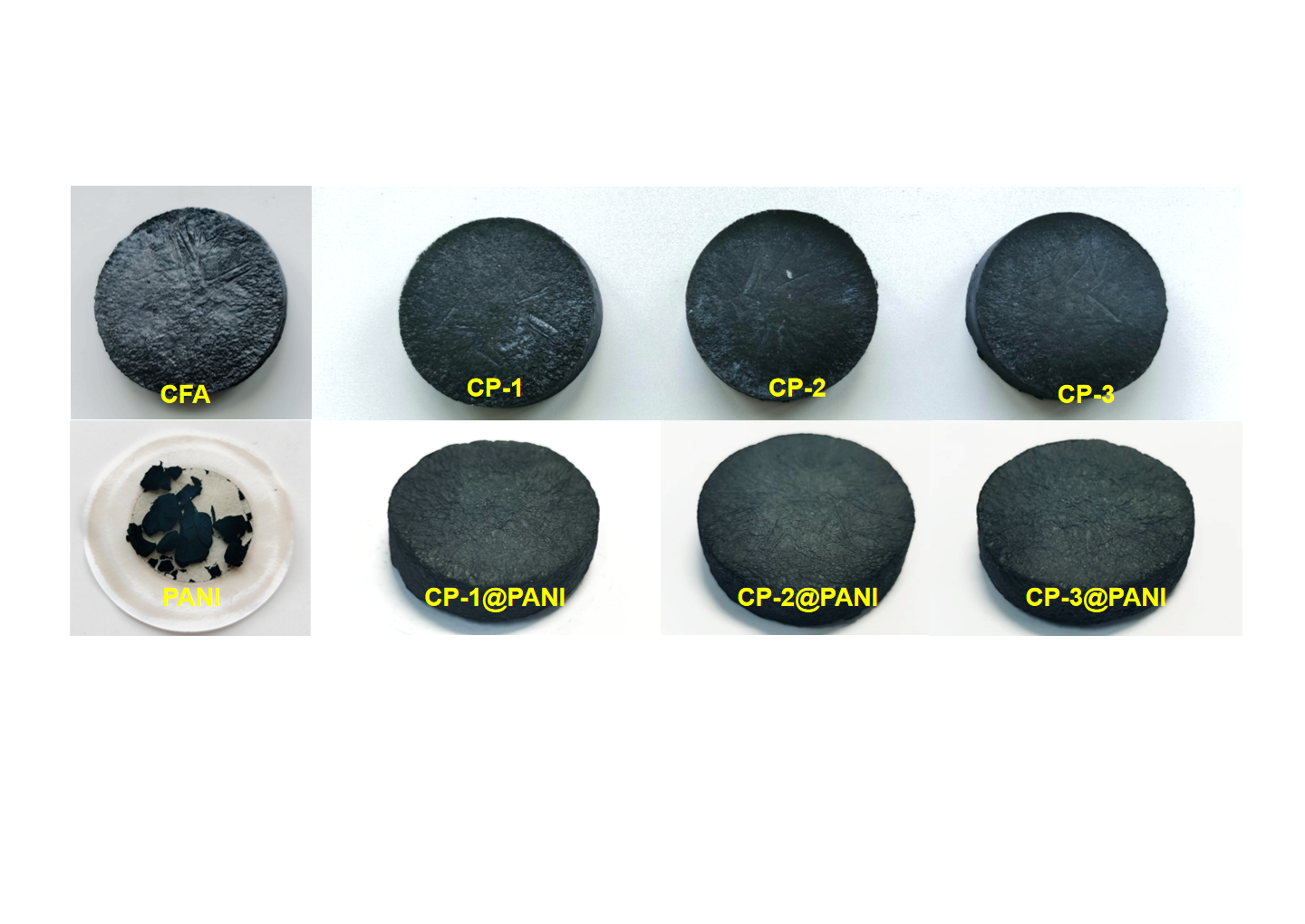
**

**Fig. S8** Photographs of the color change of the aerogels and the precipitated PANI particles


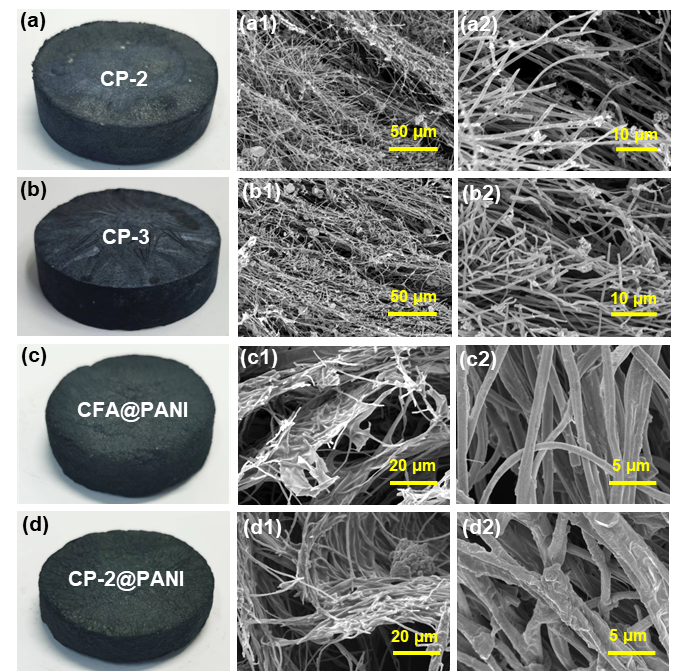


**Fig. S9** Photographs and cross-sectional SEM images of **a** CP-2 aerogel. **b** CP-3 aerogel **c** CFA@PANI aerogel. **d** CP-2@PANI aerogel


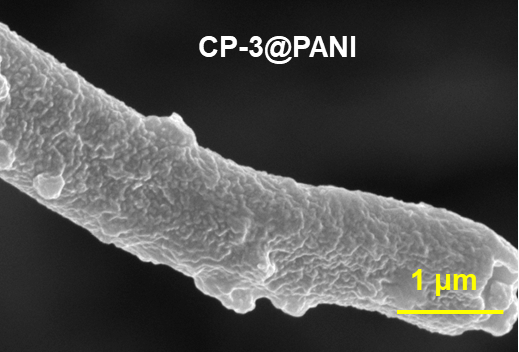


**Fig. S10** SEM image of CP-3@PANI aerogel


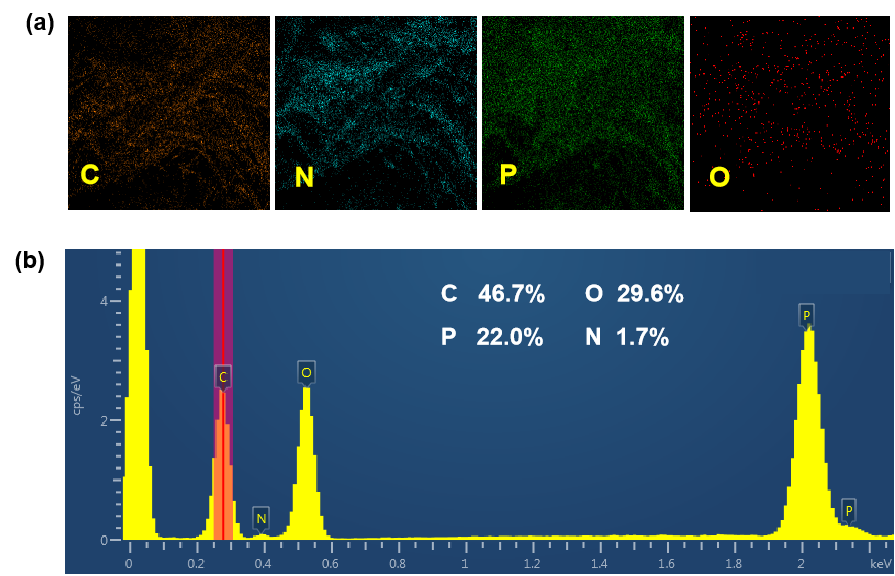


**Fig. S11** EDS mapping of C, N, O, P elements and their proportion of the CP-3@PANI

**Fig. S12** Raman spectra of CFA, PANI Power and CNFs/PANI hybrid aerogels


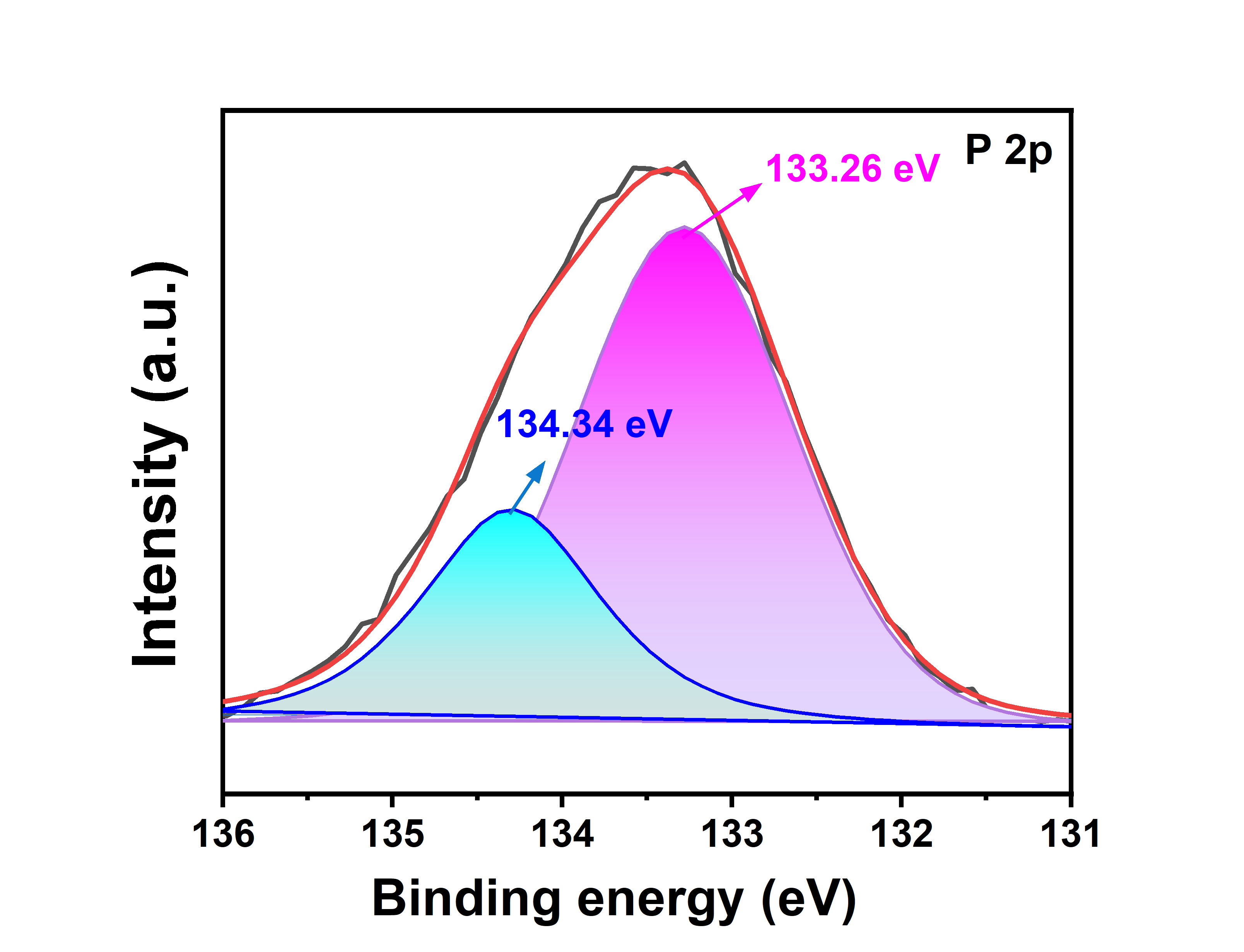


**Fig. S13** P 2p spectrum of CP-3@PANI


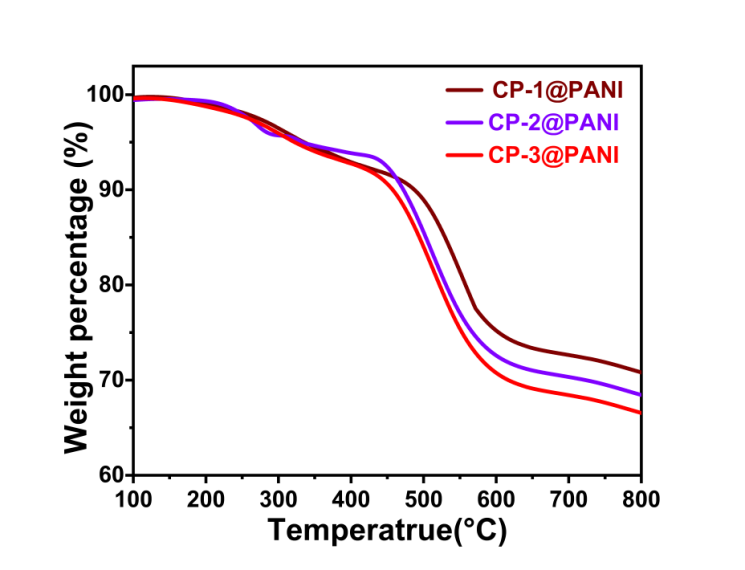


**Fig. S14** TGA curves of CP-1@PANI, CP-2@PANI, and CP-3@PANI


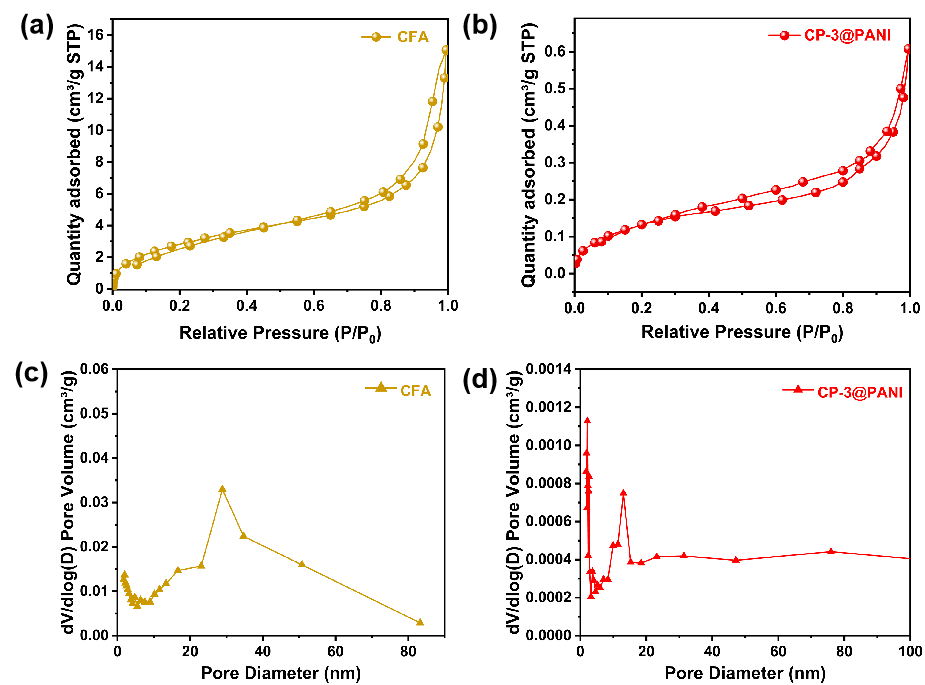


**Fig. S15** N_2_ adsorption–desorption isotherms and pore size distribution curves of CFA and CP-3@PANI


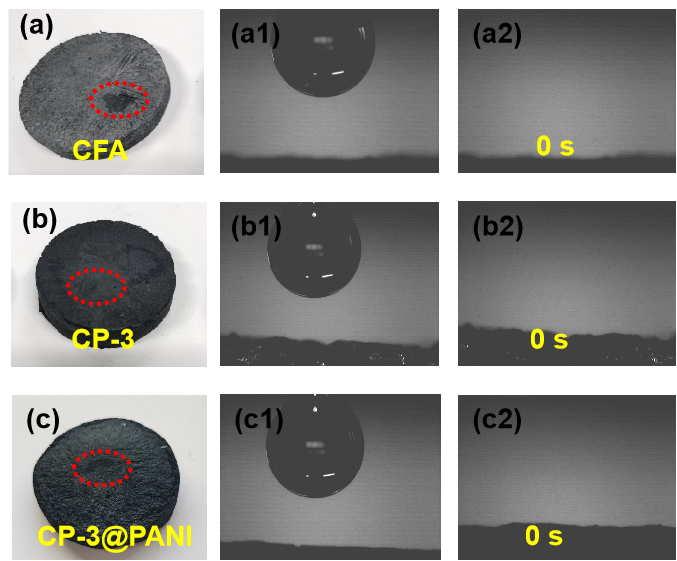


**Fig. S16** Water contact angle (WCA) of the CFA, CP-3, and CP-3@PANI


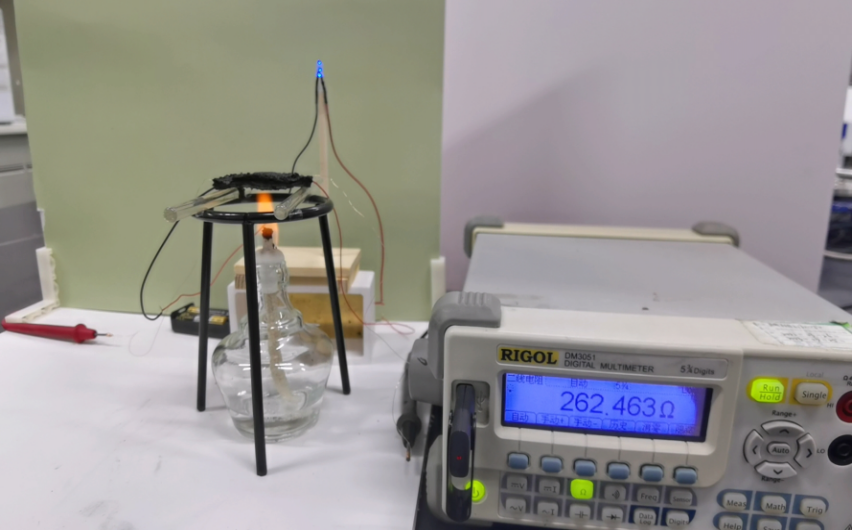


**Fig. S17** Construction of a device for testing the resistance change of CP-3@PANI aerogel during heat treatment


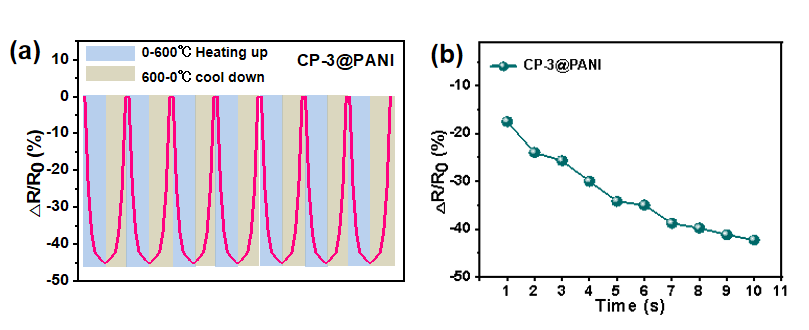


**Fig. S18 a** Temperature-resistance dependence of CP-3@PANI during heating-cooling cycle; **b** Time-resistance change rate curve of CP-3@PANI during heat treatment

**Fig. S19** Conductivity of PANI doped with different acids in CP-3@PANI


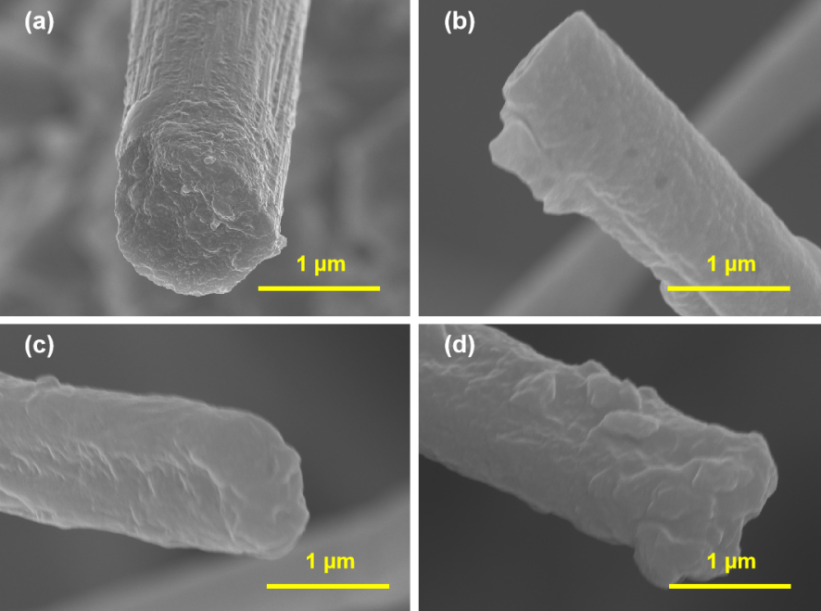


**Fig. S20** SEM images of CP-3@PANI aerogels in-situ polymerization for 4h, 8h, 12h, and 16h


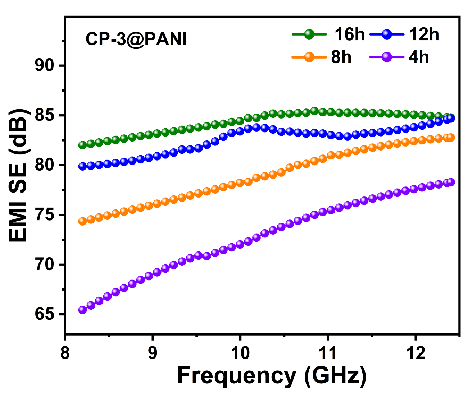


**Fig. S21** EMI SE of CP-3@PANI aerogels in-situ polymerization for 4h, 8h, 12h, and 16h


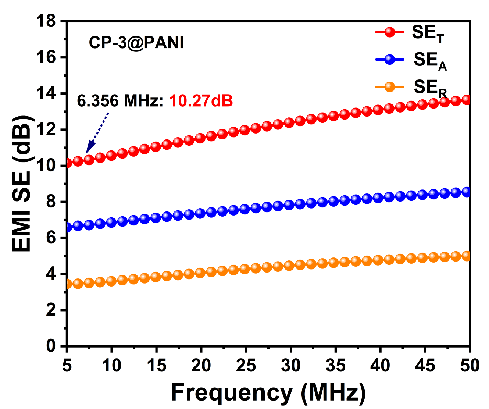


**Fig. S22** SE_T_, SE_R_, and SE_A_ values of CP-3@PANI in the 5-50 MHz


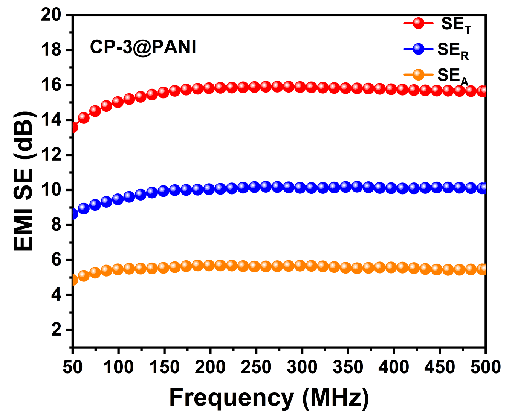


**Fig. S23** SE_T_, SE_R_, and SE_A_ values of CP-3@PANI in the 50-500 MHz


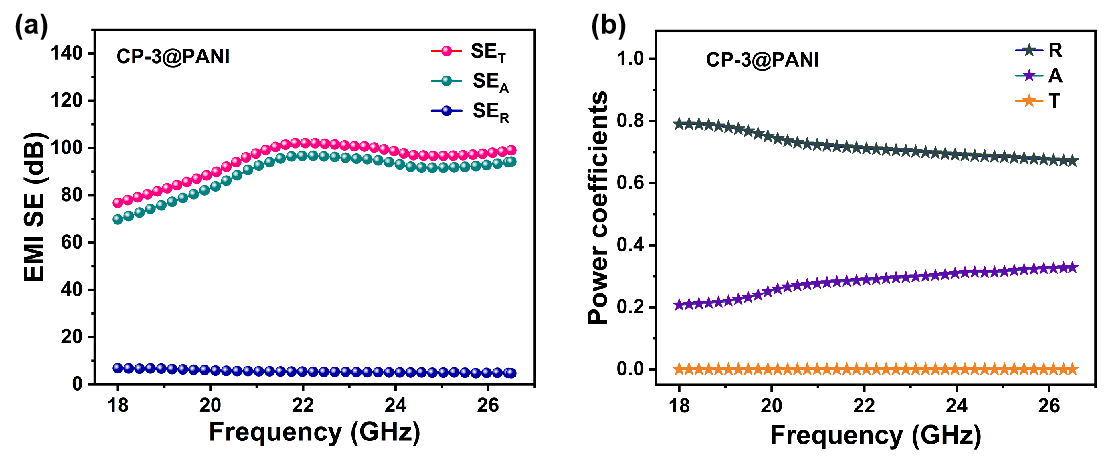


**Fig. S24** (**a)** the SE_T_, SE_R_, and SE_A_ value of CP-3@PANI in the K-band. (**b)** the R, T, and A value of CP-3@ PANI in the K-band


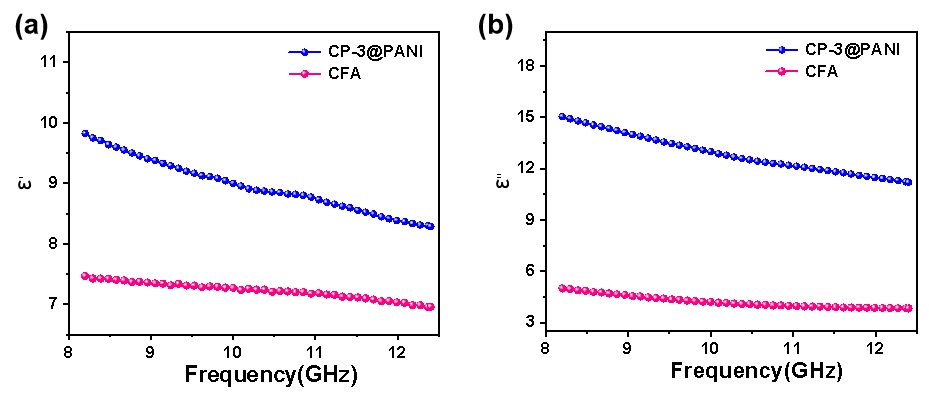


**Fig. S25** (**a**) real part ε' and (**b**) imaginary part ε“of CFA and CP-3PANI


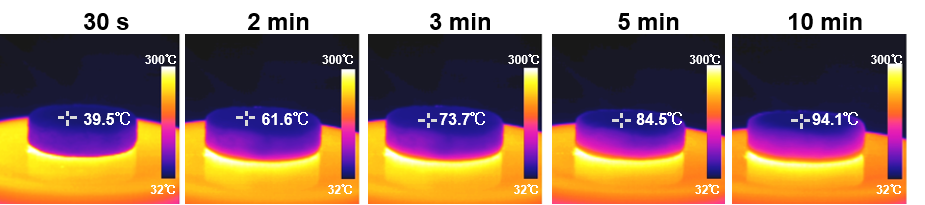


**Fig. S26** Thermal infrared images of the CP-3@PANI on a 300 °C heating platform after 10 repeated heating cycles


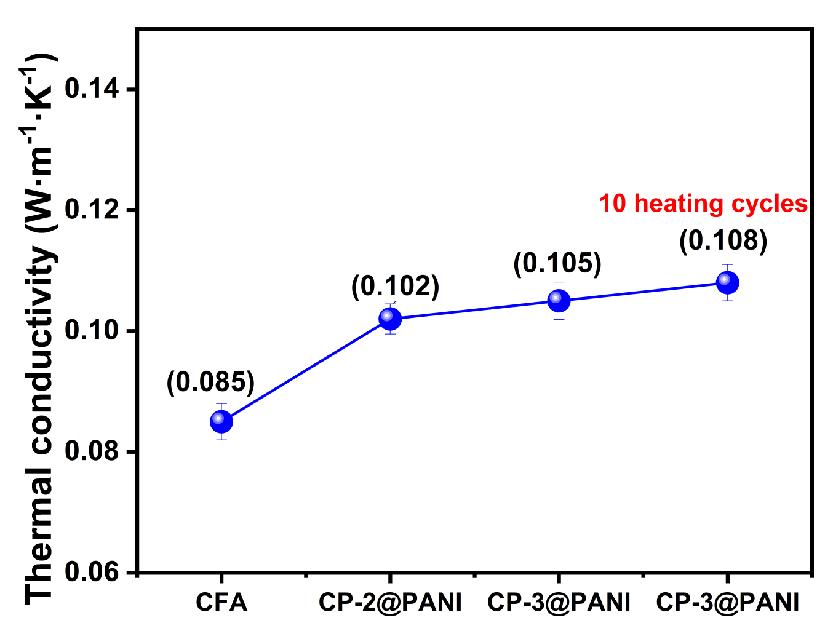


**Fig. S27** Thermal conductivity of CFA, CP-2@PANI, CP-3@PANI and CP-3@PANI after 10 repeated heating cycles


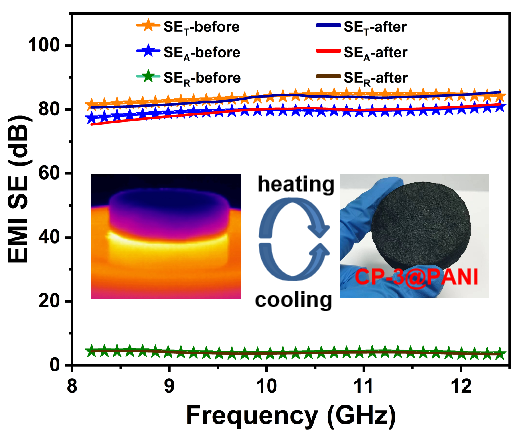


**Fig. S28** EMI SE values of the CP-3@PANI after 10 heating and cooling cycles (from RT-300 °C)


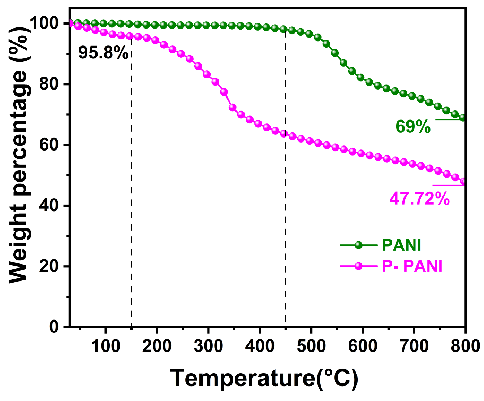


**Fig. S29** TGA curves of P-PANI and PANI


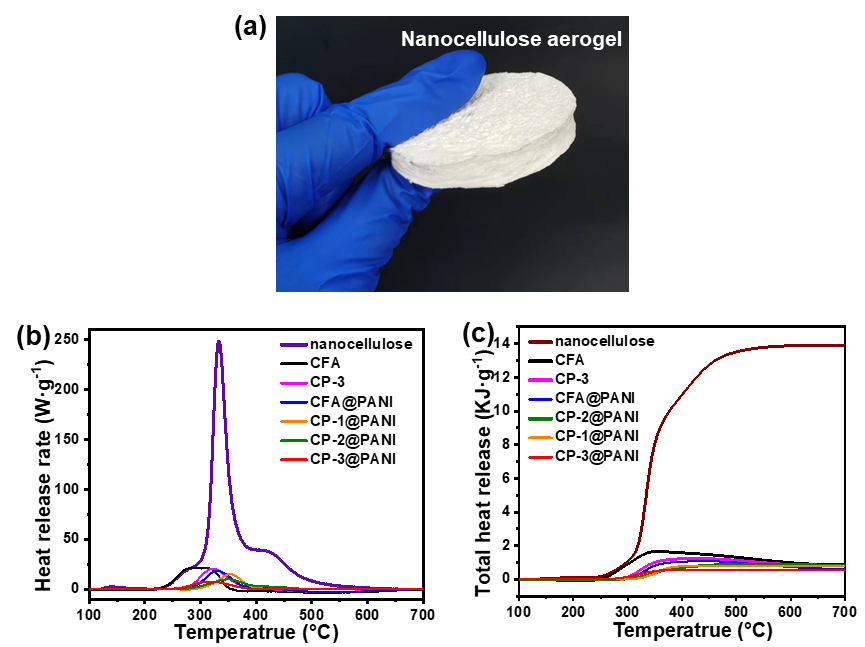


**Fig. S30 a** Photo of nanocellulose aerogel; **b** HHR curves of the nanocellulose aerogel and composite aerogels; **c** THR curves of the nanocellulose aerogels and composite aerogels


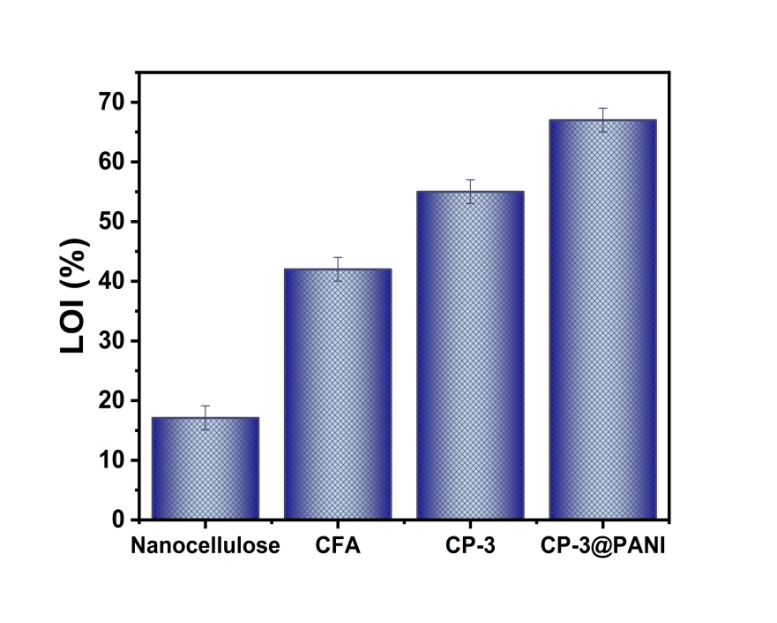


**Fig. S31** LOI values of the pure nanocellulose, CFA, CP-3 and CP-3@PANI aerogel

**Table S1** The compositions of the CNFs/PANI aerogels

| **Samples** | **Carbon nanofiber (g)** | **PANI**  **(g)** | **CNFs : PANI**  **(g)** | **Nanocellulose**  **Suspension (g)^a^** | **Density**  **(g cm^-3^)** |
| --- | --- | --- | --- | --- | --- |
| CFA | 0.32 | 0 | 1:0 | 4 | 0.01 |
| CP-1 | 0.32 | 0.32g | 1:1 | 4 | 0.02 |
| CP-2 | 0.32 | 0.64g | 1:2 | 4 | 0.034 |
| CP-3 | 0.32 | 0.96g | 1:3 | 4 | 0.043 |

1. Weight concentration of the nanocellulose suspension is 1.5 wt%.

**Table S2** Some Typical Data for Fiber Shell Polymerization

| **Sample** | **Weight of ANI**  **(g)** | **Weight increase of the sample (g)** | **% Yield of PANI^b^ (only on the**  **aerogel)** | **Conductivity (S/m)** | **Density (g/cm^3^)** | **PA content (wt%)** |
| --- | --- | --- | --- | --- | --- | --- |
| CFA@PANI | 1 | 0.54 | 11.9 | 100 | 0.075 | 17 |
| CP-1@PANI | 1 | 0.63 | 13.9 | 120 | 0.08 | 21 |
| CP-2@PANI | 1 | 0.75 | 16.6 | 170 | 0.095 | 22.7 |
| CP-3@PANI | 1 | 0.82 | 18.2 | 199 | 0.108 | 23.8 |

b) Yield of the PANI salts was calculated based on the amount of aniline used in the reaction.

**Table S3** Surface area, pore volume, and average pores size of the CFA and CP-3@PANI

| **Sample** | **S_BET_ (m^2^·g^-1^)** | **Pore volume (cm^3^·g^-1^)** | **Pore size (nm)** |
| --- | --- | --- | --- |
| CFA | 10.98 | 0.023 | 9.053 |
| CP-3@PANI | 0.52 | 0.000976 | 7.45 |

**Table S4** The sample parameters and SSE/d values of carbon-based and PANI-based aerogel

| **Materials** | **Density**  **[g·cm^-3^]** | **Frequency**  **[GHz]** | **EMI SE**  **[dB]** | **SSE/d**  **[dB· cm^3^ ·g^-1^]** | **Refs.** |
| --- | --- | --- | --- | --- | --- |
| PANI/MWCNT/graphene | 0.2 | 12.4-18 | 42 | 196 | [S1] |
| LSW/PVA/PANI | 0.55 | 8.2-12.4 | 40 | 73 | [S2] |
| Wood-derived carbon | 0.072 | 8.2-12.4 | 25.5 | 354 | [S3] |
| HfC-carbon fibers/graphene | 0.18 | 8.2-12.4 | 66.4 | 364.1 | [S4] |
| NbC-pyrolytic carbon | 0.48 | 8.2-12.4 | 54.8 | 114 | [S5] |
| Carbon foam | 0.15 | 8.2-12.4 | 53 | 353 | [S6] |
| Carbon-rGO | 0.31 | 8.2-12.2 | 50 | 161 | [S7] |
| GRA Carbon foam | 0.166 | 8.2-12.4 | 40 | 241 | [S8] |
| Carbon/PN resin | 0.152 | 8.2-12.4 | 51.2 | 341 | [S9] |
| Carbon fiber/nano copper | 0.317 | 8.2-12.4 | 29.3 | 92 | [S10] |
| Aerogel-like Carbon | 0.121 | 8.2-12.4 | 51 | 421 | [S11] |
| Graphene-carbon | 0.07 | 8.2-12.4 | 37 | 528 | [S12] |
| WTP-PVA Carbon | 0.057 | 8.2-12.4 | 40 | 700 | [S13] |
| Phthalonitrile-Based Carbon | 0.15 | 8.2-12.4 | 51.2 | 341.1 | [S14] |
| Sugarcane-derived carbon | 0.112 | 8.2-12.4 | 51 | 455.4 | [S15] |
| Bread-derived carbon | 0.29 | 8.2-12.4 | 17.2 | 59.3 | [S16] |
| RF/MWCNTs/Fe3O4 carbon | 0.125 | 8.2-12.4 | 62 | 493.6 | [S17] |
| Carbon/MWCNTs | 0.52 | 8.2-12.4 | 85 | 163 | [S18] |
| Wood-derived carbon/AgNWs | 0.13 | 8.2-12.4 | 60 | 465.1 | [S19] |
| Carbon honeycomb/rGO | 0.061 | 8.2-12.4 | 42 | 688.5 | [S20] |
| 3D-carbon texture/rGO | 0.07 | 8.2-12.4 | 37 | 528 | [S21] |
| Carbon foam | 0.121 | 8.2-12.4 | 20 | 165 | [S22] |
| **CP-3@PANI** | **0.108** | **8.2-12.4** | **85.45** | **791.2** | **This work** |

**Table S5** Related MCC Data of CNF aerogel

| **Sample** | **PHRR (W g^-1^)** | **T*p* (℃)** | **THR (kJ g^-1^)** | **HRC (J g^-1^ K^-1^)** |
| --- | --- | --- | --- | --- |
| CNF | 248.9 | 322.7 | 13.9 | 248.4 |

**Table S6** CF-3@PANI Comparison of comprehensive performance with samples reported in literatures

| **Materials** | **EMI SE**  **[dB]** | **Thermal conductivity (W·m^-1^·K^-1^)** | **PHHR**  **[W·g^-1^]** | **Refs.** |
| --- | --- | --- | --- | --- |
| Carbon foams | 38.9 | 0.185 |  | [S23] |
| rGO/sugarcane derived hybrid carbon foam | 53 | 0.115 |  | [S24] |
| PCC/MXene/polyvinyl alcohol | 43.13 | 0.69 | 207.6 | [S25] |
| MXene/polyphosphamide/PA6 | 24 |  | 300 | [S26] |
| SPI/PA/GNS-co-Q | 43 |  | 315.46 | [S27] |
| (APP)/PEI/MXene/(PCL) ramie fabric | 35 |  | 66.3 | [S28] |
| UPPH-SMCs | 77 |  | 83 | [S29] |
| FRs/CNT/TPU | 32.7 |  | 28.4 | [S30] |
| PS/SiAPP/MWCNT | 11 |  | 399 | [S31] |
| P-doped PANI/AgMWs | 51.2 |  | 223 | [S32] |
| Melamine-Based MF/Fe3O4/AgNWs | 49 |  | 75.9 | [S33] |
| CoFe2O4@CNT/PDMS Foams | 48.85 | 0.106 |  | [S34] |
| MXene/CNT/Epoxy | 34.7 |  | 532 | [S35] |
| MXene/montmorillonite/CNF | 43 | 0.159 |  | [S36] |
| F-reduced graphene oxide (rGO)/paraffin | 74.6 | 0.36 | 534 | [S37] |
| PS-MWCNT-IFR | 38 |  | 171 | [S38] |
| **CP-3@PANI** | **85.45** | **0.104** | **7.8** | **This work** |

**Supplementary References**

1. Y. Huangfu, K. Ruan, H. Qiu, Y. Lu, C. Liang et al., Fabrication and investigation on the PANI/MWCNT/thermally annealed graphene aerogel/epoxy electromagnetic interference shielding nanocomposites. Composites, Part A. **121**, 265-272 (2019). <https://doi.org/10.1016/j.compositesa.2019.03.041>
2. T. Zhang, S. Zeng, H. Jiang, Z. Li, D. Bai et al., Leather solid waste/poly(vinyl alcohol)/polyaniline aerogel with mechanical robustness, flame retardancy, and enhanced electromagnetic interference shielding. ACS Appl Mater Interfaces **13**(9), 11332-11343 (2021). <https://doi.org/10.1021/acsami.1c00880>
3. X. Liu, Y. Li, X. Sun, W. Tang, G. deng et al., Off/on switchable smart electromagnetic interference shielding aerogel. Matter. **4**, (2021). <https://doi.org/10.1016/j.matt.2021.02.022>
4. D. Jiang, S. Tian, H. Li, Z. Du, T. Liu et al., Lightweight hfc nanowire-carbon fiber/graphene aerogel composites for high-efficiency electromagnetic interference shielding. Carbon. **219**, 118788 (2024). [https://doi.org/10.1016/j.carbon.2024.118788](https://doi.org/https:/doi.org/10.1016/j.carbon.2024.118788)
5. X. Liu, H. Liu, H. Xu, W. Xie, M. Li et al., Natural wood templated hierarchically cellular nbc/pyrolytic carbon foams as stiff, lightweight and high-performance electromagnetic shielding materials. J. Colloid Interface Sci. **606**, 1543-1553 (2022). [https://doi.org/10.1016/j.jcis.2021.08.110](https://doi.org/https:/doi.org/10.1016/j.jcis.2021.08.110)
6. A. Chithra, P. Wilson, S. Vijayan, R. Rajeev, K. Prabhakaran. Carbon foams with low thermal conductivity and high emi shielding effectiveness from sawdust. Industrial Crops and Products. **145**, 112076 (2020). [https://doi.org/10.1016/j.indcrop.2019.112076](https://doi.org/https:/doi.org/10.1016/j.indcrop.2019.112076)
7. P. R. Agrawal, R. Kumar, S. Teotia, S. Kumari, D. P. Mondal et al., Lightweight, high electrical and thermal conducting carbon-rgo composites foam for superior electromagnetic interference shielding. Composites, Part B. **160**, 131-139 (2019). [https://doi.org/10.1016/j.compositesb.2018.10.033](https://doi.org/https:/doi.org/10.1016/j.compositesb.2018.10.033)
8. F. Moglie, D. Micheli, S. Laurenzi, M. Marchetti, V. Mariani Primiani. Electromagnetic shielding performance of carbon foams. Carbon. **50**(5), 1972-1980 (2012). [https://doi.org/10.1016/j.carbon.2011.12.053](https://doi.org/https:/doi.org/10.1016/j.carbon.2011.12.053)
9. E. Zhou, J. Xi, Y. Guo, Y. Liu, Z. Xu et al., Synergistic effect of graphene and carbon nanotube for high-performance electromagnetic interference shielding films. Carbon. **133**, 316-322 (2018). [https://doi.org/10.1016/j.carbon.2018.03.023](https://doi.org/https:/doi.org/10.1016/j.carbon.2018.03.023)
10. Y. Jiao, C. Wan, W. Zhang, W. Bao, J. Li. Carbon fibers encapsulated with nano-copper: A core‒shell structured composite for antibacterial and electromagnetic interference shielding applications. Nanomaterials (Basel). **9**(3), (2019). <https://doi.org/10.3390/nano9030460>
11. Y.-Q. Li, Y. A. Samad, K. Polychronopoulou, K. Liao, Lightweight and highly conductive aerogel-like carbon from sugarcane with superior mechanical and emi shielding properties. ACS Sustainable Chem. Eng. **3**(7), 1419-1427 (2015). <https://doi.org/10.1021/acssuschemeng.5b00340>
12. W.-L. Song, X.-T. Guan, L.-Z. Fan, W.-Q. Cao, C.-Y. Wang et al., Tuning three-dimensional textures with graphene aerogels for ultra-light flexible graphene/texture composites of effective electromagnetic shielding. Carbon. **93**, 151-160 (2015). [https://doi.org/10.1016/j.carbon.2015.05.033](https://doi.org/https:/doi.org/10.1016/j.carbon.2015.05.033)
13. L. Vazhayal, P. Wilson, K. Prabhakaran, Waste to wealth: Lightweight, mechanically strong and conductive carbon aerogels from waste tissue paper for electromagnetic shielding and CO_2_ adsorption. Chem. Eng. J. **381**, 122628 (2020). [https://doi.org/10.1016/j.cej.2019.122628](https://doi.org/https:/doi.org/10.1016/j.cej.2019.122628)
14. L. Zhang, M. Liu, E. Chua, K. Y. See, X. Hu, Phthalonitrile-based carbon foam with high specific mechanical strength and superior electromagnetic interference shielding performance. ACS Appl. Mater. Interfaces **8**, 5b12072 (2016). <https://doi.org/10.1021/acsami.5b12072>
15. Y. Zhang, J. Yu, J. Lu, C. Zhu, D. Qi, Facile construction of 2d mxene (ti3c2tx) based aerogels with effective fire-resistance and electromagnetic interference shielding performance. J. Alloys Compd. **870**, 159442 (2021). [https://doi.org/10.1016/j.jallcom.2021.159442](https://doi.org/https:/doi.org/10.1016/j.jallcom.2021.159442)
16. Y. Yuan, Y. Ding, C. Wang, F. Xu, Z. Lin et al., Multifunctional stiff carbon foam derived from bread. ACS Appl. Mater. Interfaces **8**(26), 16852-16861 (2016). <https://doi.org/10.1021/acsami.6b03985>
17. Q. Li, L. Chen, J. Ding, J. Zhang, X. Li et al., Open-cell phenolic carbon foam and electromagnetic interference shielding properties. Carbon **104**, 90-105 (2016). [https://doi.org/10.1016/j.carbon.2016.03.055](https://doi.org/https:/doi.org/10.1016/j.carbon.2016.03.055)
18. R. Kumar, S. R. Dhakate, T. Gupta, P. Saini, B. P. Singh et al., Effective improvement of the properties of light weight carbon foam by decoration with multi-wall carbon nanotubes. J. Mater. Chem. A **1**(18), 5727-5735 (2013). <https://doi.org/10.1039/C3TA10604G>
19. Y. Yuan, X. Sun, M. Yang, F. Xu, Z. Lin et al., Stiff, thermally stable and highly anisotropic wood-derived carbon composite monoliths for electromagnetic interference shielding. ACS Appl. Mater. Interfaces **9**(25), 21371-21381 (2017). <https://doi.org/10.1021/acsami.7b04523>
20. Y. Yuan, L. Liu, M. Yang, T. Zhang, F. Xu et al., Lightweight, thermally insulating and stiff carbon honeycomb-induced graphene composite foams with a horizontal laminated structure for electromagnetic interference shielding. Carbon **123**, 223-232 (2017). [https://doi.org/10.1016/j.carbon.2017.07.060](https://doi.org/https:/doi.org/10.1016/j.carbon.2017.07.060)
21. W.-L. Song, X.-T. Guan, L.-Z. Fan, W.-Q. Cao, C.-Y. Wang et al., Tuning three-dimensional textures with graphene aerogels for ultra-light flexible graphene/texture composites of effective electromagnetic shielding. Carbon **93**, 151 (2015). <https://doi.org/10.1016/j.carbon.2015.05.033>
22. W. Li, L. Feng, X. Shi, Y. Wang, Mechanical and electromagnetic shielding properties of carbon foam. Adv. Engin. Mater. **23**, 2100452 (2021). <https://doi.org/10.1002/adem.202100452>
23. A. Chithra, P. Wilson, S. Vijayan, R. Rajeev, K. Prabhakaran, Thermally insulating robust carbon composite foams with high emi shielding from natural cotton. J. Mater. Sci. Technol. **94**, 113-122 (2021). [https://doi.org/10.1016/j.jmst.2021.02.064](https://doi.org/https:/doi.org/10.1016/j.jmst.2021.02.064)
24. L. Wang, X. Shi, J. Zhang, Y. Zhang, J. Gu, Lightweight and robust rgo/sugarcane derived hybrid carbon foams with outstanding EMI shielding performance. J. Mater. Sci. Technol. **52**, 119-126 (2020). [https://doi.org/10.1016/j.jmst.2020.03.029](https://doi.org/https:/doi.org/10.1016/j.jmst.2020.03.029)
25. S. Gong, X. Sheng, X. Li, M. Sheng, H. Wu et al., A multifunctional flexible composite film with excellent multi‐source driven thermal management, electromagnetic interference shielding, and fire safety performance, inspired by a “brick–mortar” sandwich structure. Adv. Funct. Mater. **32**，2200570 (2022). <https://doi.org/10.1002/adfm.202200570>
26. V. K. Patle, Y. Mehta, N. Dwivedi, D. P. Mondal, A. K. Srivastava et al., Thermal insulating and fire-retardant lightweight carbon-slag composite foams towards absorption dominated electromagnetic interference shielding. Sustainable Mater. Technol. **33,** e00453 (2022). [https://doi.org/10.1016/j.susmat.2022.e00453](https://doi.org/https:/doi.org/10.1016/j.susmat.2022.e00453)
27. J. Liu, J. Zhang, F. Chen, X. Cui, P. Qi et al., Enhancing fire safety, electromagnetic interference shielding, and photothermal conversion performances of wearable polyamide fabrics through eco-friendly coatings. Sustainable Mater. Technol. **40**, e00949 (2024). [https://doi.org/10.1016/j.susmat.2024.e00949](https://doi.org/https:/doi.org/10.1016/j.susmat.2024.e00949)
28. W. Cheng, Y. Zhang, Y. Tao, J. Lu, J. Liu et al., Durable electromagnetic interference (emi) shielding ramie fabric with excellent flame retardancy and self-healing performance. J. Colloid Interface Sci. **602**, 810-821 (2021). <https://doi.org/10.1016/j.jcis.2021.05.159>
29. C.-C. Höhne, P. Blaess, S. Ilinzeer, P. Griesbaum, New approach for electric vehicle composite battery housings: Electromagnetic shielding and flame retardancy of pur/up-based sheet moulding compound. Composites, Part A. **167**, 107404 (2023). [https://doi.org/10.1016/j.compositesa.2022.107404](https://doi.org/https:/doi.org/10.1016/j.compositesa.2022.107404)
30. X. Ji, D. Chen, J. Shen, S. Guo, Flexible and flame-retarding thermoplastic polyurethane-based electromagnetic interference shielding composites. Chem. Eng. J. **370**, 1341-1349 (2019). [https://doi.org/10.1016/j.cej.2019.03.293](https://doi.org/https:/doi.org/10.1016/j.cej.2019.03.293)
31. C. Gao, Y. Shi, Y. Chen, S. Zhu, Y. Feng et al., Constructing segregated polystyrene composites for excellent fire resistance and electromagnetic wave shielding. J. Colloid Interface Sci. **606**, 1193-1204 (2022). [https://doi.org/10.1016/j.jcis.2021.08.091](https://doi.org/https:/doi.org/10.1016/j.jcis.2021.08.091)
32. L. Zhang, B.-W. Liu, Y.-Z. Wang, T. Fu, H.-B. Zhao, P-doped pani/agmws nano/micro coating towards high-efficiency flame retardancy and electromagnetic interference shielding. Composites, Part B **238**, 109944 (2022). [https://doi.org/10.1016/j.compositesb.2022.109944](https://doi.org/https:/doi.org/10.1016/j.compositesb.2022.109944)
33. H.-G. Shi, H.-B. Zhao, B.-W. Liu, Y.-Z. Wang, Multifunctional flame-retardant melamine-based hybrid foam for infrared stealth, thermal insulation, and electromagnetic interference shielding. ACS Appl. Mater. Interfaces **13**(22), 26505-26514 (2021). <https://doi.org/10.1021/acsami.1c07363>
34. Z. Xie, Z. Wei, Y. Meng, N. Pan, R. Zhang et al., Robust CoFe_2_O_4_@carbon nanotube/polydimethylsiloxane foams with low thermal conductivity for electromagnetic interference shielding. ACS Appl. Nano Mater. **6**(23), 21733-21740 (2023). <https://doi.org/10.1021/acsanm.3c03945>
35. R. Chang, H. Gao, P. Hao, H. Qu, J. Xu et al., Hierarchically porous mxene/carbon nanotube/epoxy composites for effective electromagnetic interference shielding and flame retardancy. ACS Appl. Nano Mater. **7**(3), 3168-3178 (2024). <https://doi.org/10.1021/acsanm.3c05512>
36. D.-D. Li, X. Pu, P. Hu, M. Han, W. Xin et al., Multifunctional Ti_3_C_2_T_x_ mxene/montmorillonite/cellulose nanofibril films for electromagnetic interference shielding, photothermal conversion, and thermal insulation. Cellulose **30**(6), 3793-3805 (2023). <https://doi.org/10.1007/s10570-023-05119-6>
37. Y.-R. Li, Y.-M. Li, W.-J. Hu, D.-Y. Wang. Shaped photothermal conversion phase‐change materials with excellent electromagnetic shielding performance and flame retardancy. Adv. Eng. Mater. **25**((2023). <https://doi.org/10.1002/adem.202201885>
38. D. Ponnamma, K. K. Sadasivuni, M. Strankowski, Q. Guo, S. Thomas, Synergistic effect of multi walled carbon nanotubes and reduced graphene oxides in natural rubber for sensing application. Soft Matter. **9**(43), 10343-10353 (2013). <https://doi.org/10.1039/C3SM51978C>
